# Criticality Creates a Functional Platform for Network Transitions Between Internal and External Processing Modes in the Human Brain

**DOI:** 10.3389/fnsys.2021.657809

**Published:** 2021-11-25

**Authors:** Minkyung Kim, Hyoungkyu Kim, Zirui Huang, George A. Mashour, Denis Jordan, Rüdiger Ilg, UnCheol Lee

**Affiliations:** ^1^Department of Anesthesiology, University of Michigan Medical School, Ann Arbor, MI, United States; ^2^Center for Consciousness Science, University of Michigan Medical School, Ann Arbor, MI, United States; ^3^Neuroscience Graduate Program, University of Michigan, Ann Arbor, MI, United States; ^4^Applied Mathematics and Statistics, University of Applied Sciences Northwestern Switzerland, Muttenz, Switzerland; ^5^Department of Anesthesiology, Klinikum rechts der Isar, Technische Universität München, Munich, Germany; ^6^Department of Neurology, Klinikum rechts der Isar, Technische Universität München, Munich, Germany

**Keywords:** criticality, consciousness, oscillator model, EEG, fMRI, brain network transition, information processing

## Abstract

Continuous switching between internal and external modes in the brain appears important for generating models of the self and the world. However, how the brain transitions between these two modes remains unknown. We propose that a large synchronization fluctuation of brain networks, emerging only near criticality (i.e., a balanced state between order and disorder), spontaneously creates temporal windows with distinct preferences for integrating the network’s internal information or for processing external stimuli. Using a computational model, electroencephalography (EEG) analysis, and functional magnetic resonance imaging (fMRI) analysis during alterations of consciousness in humans, we report that synchronized and incoherent networks, respectively, bias toward internal and external information with specific network configurations. In the brain network model and EEG-based network, the network preferences are the most prominent at criticality and in conscious states associated with the bandwidth 4−12 Hz, with alternating functional network configurations. However, these network configurations are selectively disrupted in different states of consciousness such as general anesthesia, psychedelic states, minimally conscious states, and unresponsive wakefulness syndrome. The network preference for internal information integration is only significant in conscious states and psychedelic states, but not in other unconscious states, suggesting the importance of internal information integration in maintaining consciousness. The fMRI co-activation pattern analysis shows that functional networks that are sensitive to external stimuli–such as default mode, dorsal attentional, and frontoparietal networks–are activated in incoherent states, while insensitive networks, such as global activation and deactivation networks, are dominated in highly synchronized states. We suggest that criticality produces a functional platform for the brain’s capability for continuous switching between two modes, which is crucial for the emergence of consciousness.

## Introduction

Continuous switching between internal and external modes allows neural circuits to identify the contrast between different sources of information and reduce the mismatch between them (Hinton and Mcclelland, 1987; O’Reilly, 1996). Continuous switching between two modes has been considered a functional basis at a system level for constructing inner models in the brain that support perception, prediction, and action in the external world (Hasselmo, 1995; Honey et al., 2017). However, the origin of such modes in the brain, the mechanism by which they transition, and whether these transitions represent a necessary process for supporting higher-order brain functions are unknown. In our previous computational model study, we demonstrated that the brain’s responsiveness to external stimuli depends on the level of global brain network synchronization, and this dependence only emerges near a critical state (Kim and Lee, 2020). In this study, we expanded the previous computational model study, suggesting that a large synchronization fluctuation emerging near a critical state may produce a functional platform upon which functional brain networks fluctuate between two distinct modes, one of which is conducive to the integration of internal information in the network and the other of which is highly susceptible to external stimuli. Such distinct preferences for internal or external information originate from the general property of the network’s responsiveness to the synchronization fluctuation. We also analyzed both high-density electroencephalogram (EEG) and functional magnetic resonance imaging (fMRI) data of various states of consciousness (conscious, anesthetized, psychedelic, and pathological) to investigate how the network’s preference for internal or external information in the time domain is associated with different states of consciousness.

Recent computational modeling and empirical studies suggest that consciousness occurs when brain dynamics are near criticality (i.e., poised at the border of multiple states) and that losing criticality (i.e., after a transition to one of the possible states) is related to various altered states of consciousness (Kitzbichler et al., 2009; Tagliazucchi et al., 2012; Haimovici et al., 2013; Muñoz, 2018; Kim and Lee, 2019). Critical dynamics confer biological advantages that may establish a functional foundation for the emergence of consciousness: an optimal balance between stability and instability, optimal computational capability, flexibility to adapt to a changing environment, and wide repertoires of brain states (Beggs, 2008; Cocchi et al., 2017). In both biological and non-biological systems, a large global fluctuation is one of the most representative signal characteristics of criticality, with an increase in autocorrelation (Scheffer et al., 2009; Van De Leemput et al., 2014). In our previous brain network modeling study, we found that a large synchronization fluctuation near a critical state is associated with a highly variable brain sensitivity to external stimuli (Kim and Lee, 2020). Specifically, low and high levels of synchronization in the brain network, respectively, provide susceptible and refractory time windows to the stimuli. In addition, it has been suggested that the level of neural synchronization reflects the brain’s capability for information transmission and integration across the cortex (Fries, 2005; Bastos et al., 2015). Based on these findings, we hypothesize that the functional brain network may be highly susceptible to external stimuli but less internally integrative at low levels of synchronization. Conversely, at high levels of synchronization, the brain network favors information integration within the network but is less susceptible to external stimuli. Thus, we hypothesized that the brain network at high and low levels of network synchronization possesses distinct preferences for, respectively, internal and external information, which may induce the internal and external modes of the brain in the time domain. Such distinct preferences may be the most significant in conscious states (i.e., near criticality) with the maximal difference between high and low levels of synchronization. In contrast, these preferences may be mitigated in different states of consciousness (i.e., sub- or supercritical states) with reduced synchronization fluctuation.

**FIGURE 1:**
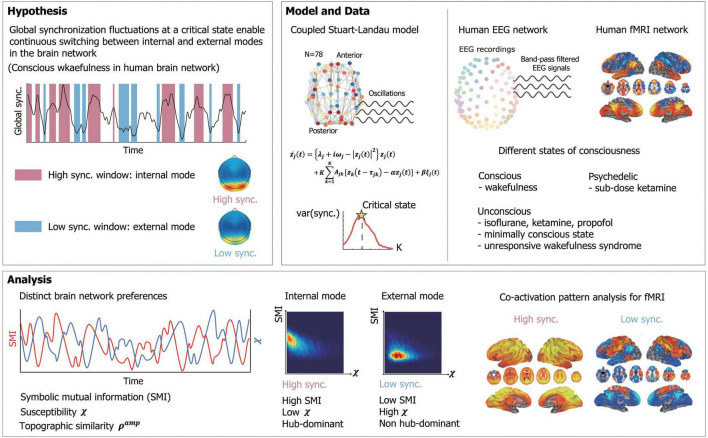
Schematic diagram of study design. We hypothesized that a large global synchronization fluctuation emerging near a critical state enables continuous switching between internal (conducive to integration of internal information) and external (highly susceptible to external stimuli) modes with distinct functional network configurations in a human brain network during conscious wakefulness. The study was composed of two parts, a mathematical modeling and an experimental analysis. We constructed a human brain network model, applying a coupled Stuart-Landau model to a human brain network structure consisting of 78 brain regions. The critical state was found with a maximum variance of the global synchronization. Human electroencephalogram (EEG) data were acquired in different states of consciousness including conscious states (CS), psychedelic state induced by sub-dose of ketamine, unconscious states (UCS) induced by isoflurane and ketamine, minimally conscious state (MCS) and unresponsive wakefulness syndrome (UWS). Functional magnetic resonance imaging (fMRI) data were acquired during CS, UCS induced by propofol and UWS. With the model and EEGs, we measured symbolic mutual information (SMI) and susceptibility χ in a sub-second time window to investigate the brain network’s preference for internal and external mode. The topographic similarity *S^amp^* was measured to observe functional network configurations for each high and low synchronization window. The co-activation pattern analysis was performed for the fMRI data to obtain fMRI networks related to internal and external modes.

To test this hypothesis, we used a large-scale human brain network model, modulating criticality and examining whether brain networks at high and low levels of synchronization near criticality have distinct preferences for the internal information of the network and external stimuli, respectively. Symbolic mutual information (SMI) (King et al., 2013) and network susceptibility (Yoon et al., 2015) were measured for each sub-second temporal window of simulated brain signals to evaluate quantitatively the brain network’s preference. The modeling results were tested in humans with high-density EEG during various states of consciousness: conscious wakefulness, anesthetized (with isoflurane and ketamine), psychedelic (with a subanesthetic-dose of ketamine), and pathological conditions such as minimally conscious states and unresponsive wakefulness syndrome. We also examined functional brain network configurations at high and low levels of synchronization that systematically contribute to those preferences. In addition, we analyzed the fMRI data during wakefulness, propofol-induced unconscious states, and unresponsiveness wakefulness syndrome to examine whether the results observed in the model and EEG data were also consistent with what we observed in the fMRI functional networks. We investigated whether the fMRI functional networks that predominate at high or low levels of synchronization are relevant to the well-known networks presumably associated with internal or external information processing. The schematic diagram of the study is presented in Figure 1. The figures from the fMRI data analysis were adapted from Huang et al. (2020).

## Materials and Methods

### Simulation of Spontaneous Neural Oscillations

A coupled Stuart-Landau model with human brain network structure has been widely used to simulate the oscillatory dynamics from various types of imaging modalities including EEG (Kim and Lee, 2019), magnetoencephalography (MEG), and fMRI (Deco et al., 2017, 2018). Here we also used the coupled Stuart-Landau model to simulate the oscillatory dynamics of brain network and investigated relationships between network synchronization and variables associated brain network’s preferences for integrating information internally in the brain network and receiving stimuli from the outside of the brain.

Spontaneous networked oscillations were generated using a coupled Stuart-Landau model in a group-averaged anatomical human brain network constructed from diffusion tensor imaging (DTI) of 78 nodes (Gong et al., 2009). The coupled Stuart-Landau model, composed of the *N* number of oscillators, is defined as the following:


$$\begin{array}{c} \dot{z_{j}}\left(t\right)=\left\{\lambda_{j}+i\omega_{j}-{\left|z_{j}\left(t\right)\right|}^{2}\right\}z_{j}\left(t\right)\\ \;+\sum_{k=1}^{N}A_{jk}K_{jk}\left\{z_{k}\left(t-\tau_{jk}\right)-\alpha z_{j}\left(t\right)\right\}+\beta \xi_{j}\left(t\right) \end{array}$$

where a complex variable *z*_*j*_(*t*) represents the oscillatory dynamics of brain region *j* at time *t*. ω_*j*_ is an initial angular natural frequency of oscillator *j*. We used Gaussian distribution for the natural frequency with a mean frequency of 10 Hz and a standard deviation of 0.5 Hz to simulate the peak frequency bandwidth of human EEG activity (Kim et al., 2018; Kim and Lee, 2020). *A*_*jk*_ = 1 if oscillators *j* and *k* are connected, and *A*_*jk*_ = 0 if they are not, based on the structural brain network. τ_*jk*_ is a time delay between oscillators *j* and *k*, defined as *D*_*jk*_/*s*, with the distance between brain regions *j* and *k*, *D*_*jk*_, and the average speed of axons in brain regions, *s*. Here we used *s=7ms*. The node *j* receives input from connected node *k* after the time delay τ_*jk*_. λ_*j*_ and *K*_*jk*_ are a bifurcation parameter of oscillator *j* and a coupling strength between oscillators *j* and *k*, respectively. Modulating these parameters induce competition of independent behavior of oscillator and the coupling among the oscillators so that complex oscillatory dynamics differently emerge in different parameter regions. Each node shows supercritical Hopf bifurcation, and the dynamics of the oscillator settle on a limit cycle if λ_*j*_ > 0, and on a stable focus if λ_*j*_ < 0. We used a homogeneous bifurcation parameter λ_*jk*_ = λ from −3.2 to 3.2 with δλ = 0.2, and a coupling strength *K*_*jk*_ = *K* from 0 to 1 with δ*K* = 0.02. We additionally modulated a diffusive coupling parameter α. The α controls the degree of outgoing flow of node *j*. In neural networks, two extreme values of α, 0 and 1 indicate two types of synapses, chemical synapses, and gap junctions, respectively. We tested 0, 0.5, and 1 for the α, and set α = 0.5 as the empirically well-fitted parameter. Further investigation for this parameter will be explored in the future study. *ξ*_*j*_(*t*) is a Gaussian white noise for each node *j* and added to the dynamics with the standard deviation β = 0.05. We numerically solved differential equations of the Stuart-Landau model using the Stratonovich-Heun method with 1,000 discretization steps, then we resampled the data with 500 Hz. The last 60 s were used for the analysis of each simulation after 15 s of saturation periods. Therefore, spontaneous oscillatory dynamics were generated for each brain region at each λ and each *K*. We repeated this simulation with one hundred different initial frequency distributions to obtain statistical stability.

### Experimental Protocol and Electroencephalography Acquisition

#### Isoflurane Anesthesia

The isoflurane data were collected from a cohort of 20 healthy volunteers (20−40 years) recorded at the University of Michigan, Ann Arbor, United States (Protocol #HUM0071578). The study has been reviewed by the Institutional Review Boards specializing in human subject research at University of Michigan. Written informed consent in accordance with the Declaration of Helsinki to participate in the study was obtained from all participants. Ten participants underwent general anesthesia. The participants in the anesthesia group initially received propofol at increasing infusion rates over three consecutive 5-min blocks (block 1: 100 μ*g*/*kg*/*min*, block 2: 200 μ*g*/*kg*/*min*, block 3: 300 μ*g*/*kg*/*min*). Responsiveness was quantified every 30 s by the response to the verbal command “Squeeze your left/right hand twice” with random allocation to left/right. Isoflurane was then administrated with air and 40% oxygen at 1.3 age-adjusted minimum alveolar concentration. The isoflurane was administrated for 3 h and discontinued.

We analyzed EEG data of 9 out of 10 subjects who underwent general anesthesia due to the availability of high-density EEG data (128-channel HydroCel nets, Net Amps 400 amplifiers; Electrical Geodesic, Inc., United States). All EEG channels were referenced to a vertex with 500 Hz sampling frequency. EEG data of 4-min of eye-closed resting state before isoflurane administration (baseline) and 4-min of periods during general anesthesia (ISO) without burst-suppression were used in the current study. The data have been published with different analyses and hypotheses (Lee et al., 2019).

#### Ketamine Anesthesia

The ketamine data were collected from 15 healthy volunteers (20−40 years) recorded at University of Michigan, Ann Arbor, with written informed consent to participate in the study. This study was approved by the University of Michigan Medical School Institutional Review Board, Ann Arbor, MI, United States (HUM00061087). The data have been published with different analyses (Lee et al., 2019). EEG data were acquired during a 5-min eyes-closed resting state before ketamine administration, subanesthetic ketamine infusion (0.5 mg/kg) over 40 min, followed by 8 mg ondansetron, break for completion of questionnaire, anesthetic (1.5 mg/kg) bolus dose, and recovery period. EEG data of 4-min eyes-closed resting state (baseline), 4-min subanesthetic ketamine-induced state (PSY), and 4-min ketamine-induced unconsciousness (KET) after bolus anesthetic administration were used in the current study. The EEG data were acquired with 128-channel HydroCel nets, Net Amps 400 amplifiers (Electrical Geodesic Inc., United States). All channels were referenced to a vertex with 500 Hz sampling frequency.

#### Disorders of Consciousness

Electroencephalography data were originally collected from a cohort of 80 patients with disorders of consciousness caused by ischemic stroke, intracerebral hemorrhage, subarachnoid hemorrhage, subdural hematoma, traumatic brain injury, meningitis, or hyperglycemic brain injury. Patients were diagnosed as minimally conscious or vegetative states/unresponsiveness wakefulness syndrome (UWS) using the Coma Recovery Scale-Revised (CRS-R). The CRS-R status was acquired again after 6 months of investigating the follow-up changes. The data from 17 subjects (the Munich cohort) were recorded on two different systems; 15 subjects were recorded with 64-channel, ring-type sintered, and nonmagnetic Ag/AgCl electrodes (Easycap, Herrsching, Germany); 2 subjects were recorded with 32-channel, nonmagnetic, and battery-operated electroencephalographic amplifiers (BrainAmp MR, Brain Products, Gilching, Germany). Both EEG data were recorded at 5 kHz sampling rates (BrainVision Recorder, Brain Products). The data from 63 patients (the Burgau cohort) were recorded with a 256-channel high-density Geodesic sensor net, a Net Amps 300 amplifier, and Net Station 4.5 software (Electrical Geodesic Inc., Eugene, OR, United States). The sampling rates were 1 kHz. All data were preprocessed to have 63-channel. In the current study, we analyzed the 4-min of the data from 9 subjects who were diagnosed as UWS without the evolution of CRS-R status and not showing suppression patterns and 16 subjects who were diagnosed as minimally conscious state (MCS).

In this study, we defined PSY as an altered state of consciousness (i.e., pharmacologically perturbed with consciousness maintained) and ISO, KET, MCS, and UWS as depressed states of consciousness (i.e., pharmacologically or pathologically perturbed with no conscious response).

### Electroencephalography Data Preprocessing

With three datasets, we analyzed 6 different states of consciousness, such as conscious state, (CS, *n* = 24), ketamine-induced psychedelic state (PSY, *n* = 15), isoflurane-induced unconsciousness (ISO, *n* = 9), ketamine-induced unconsciousness (KET, *n* = 15), MCS (*n* = 16), and UWS (*n* = 9). We selected 96-channel for the isoflurane and ketamine data and selected 56-channel for the MCS and UWS data that cover the scalp for the analysis. The MCS and UWS data were down-sampled to 250 Hz. After selecting the EEG channels, we high-pass filtered the data with 0.5 Hz cutoff frequency using MATLAB function “pop_eegfiltnew.m” in the EEGLAB toolbox. We then removed noisy epochs using MATLAB function “trimOutlier.m” with a standard deviation of channel amplitude rejection thresholds 100 μ*V*, amplitude rejection thresholds 300 μ*V*, and the range for rejection period 200-ms. Rejected channels were reconstructed using MATLAB function “pop_interp.m” in the EEGLAB with a spherical interpolation method. All EEG data from isoflurane, ketamine-induced unconsciousness, and disorders of consciousness were re-referenced to the average of all channels. We band-pass filtered the data with a frequency range from 4 to 12 Hz to capture the properties of globally coupled oscillators. We also analyzed the data with other frequency bands (1–4 Hz, 1–20 Hz, 8–12 Hz, 13–30 Hz, and 30–42 Hz).

### Level of Network Synchronization and Pair Correlation Function

In this study, the level of network synchronization is an important factor to determine the brain network’s preference for processing internal information of the network or external information from the outside of the brain network. The instantaneous level of network synchronization *r*(*t*) at time *t* was measured by the order parameter,


$$r\left(t\right)e^{i\psi \left(t\right)}=\frac{1}{N}\sum_{j=1}^{N}e^{i\theta_{j}\left(t\right)}$$

where θ_*j*_(*t*) is a phase of oscillator *j* and ψ(*t*) is the average global phase at time *t*. Here *r*(*t*) equals to 0 if phases of oscillators are uniformly distributed and 1 if all oscillators have the same phase. The *r*(*t*) was calculated for all parameter combinations in the model, all states from EEG, and fMRI data.

We then measured a pair correlation function *PCF*≡*N*[ < *r*^2^(*t*) > − < *r*(*t*) > ^2^], which is a surrogate measure of criticality, to define the critical state (Yoon et al., 2015) in the model, under the assumption that the largest synchronization fluctuations are associated with the largest number of metastable states of brain network and should be shown at criticality. We measured PCF for all parameter combinations in the model, all states of EEG data.

### Temporal Window Classification Based on the Network Synchronization

To investigate the network’s information processing preference with reliable time resolution, we classified the network transient states into high and low *R* temporal windows. We calculated an average network synchronization *R* as < *r*(*t*) > _*T*_ for each temporal window, where *T* is the size of the temporal window. In the model, we set *T* = 250 *msec* with 50 *msec* overlaps and classified the temporal windows into two different windows: one of which is the incoherent state and the other is the highly synchronized state (low and high *R* windows). In the model, we set thresholds as *R* = 0.3 and *R* = 0.5 for low and high *R* windows, respectively. We tested various threshold values such as 0.25 and 0.55, and 0.2 and 0.6, and could not find qualitative differences among them. We avoided using thresholds that were too small or too large because it eliminates low and high R time windows. For the EEG data, we set *T* = 250 *msec* with 50 *msec* overlaps for CS, PSY, ISO, and KET, and *T* = 300 *msec* with 50 *msec* overlaps for MCS and UWS due to the sampling frequency of the data. We concatenated *R* values across all temporal windows, all subjects, and all six different states of consciousness to find the total mean and standard deviation of *R*. With thresholds ${<R>}_{total}\pm 0.5\sqrt{<R^{2}\left(t\right)>-{<R\left(t\right)>}^{2}}$, we classified all temporal windows across all states into windows with low and high *R*.

### Symbolic Mutual Information and Susceptibility

To calculate the information processing capabilities for each temporal window, we measured symbolic mutual information (SMI) and network susceptibility in the model and EEG signals. The SMI measures the amount of information sharing, quantifying the extent of non-random joint fluctuations between two signals *X* and *Y*. To calculate the SMI, the signals *X* and *Y* are first transformed into sequences of discrete symbols $\left(\hat{X},\hat{Y}\right)$ with a fixed symbol size *m* with a temporal separation τ. It calculates a joint probability of co-occurring symbolic patterns between two signals.


$$SMI\left(\hat{X},\hat{Y}\right)=\frac{1}{log\left(m!\right)}\sum_{\hat{x}\in \hat{X}}\sum_{\hat{y}\in \hat{X}}p\left(\hat{x},\hat{y}\right)log\frac{p\left(\hat{x},\hat{y}\right)}{p\left(\hat{x}\right)p\left(\hat{y}\right)}$$

where $p\left(\hat{x}\right)$ is the probability occurring symbol $\hat{x}$ in the time series $\hat{X}$. We set *m* = 3, leading to 3! = 6 of different symbol pairs $\left(\hat{x},\hat{y}\right)$ that can potentially exist in the transformed symbolic time-series. In the model, we used τ = 14(28 *ms*) to get the maximum resolved frequency $f_{m}=\frac{fs}{k\times \tau }=\frac{500Hz}{3\times 14}=11.9Hz$, which is suitable for our interest of frequency range (∼12 Hz) (Imperatori et al., 2019). The SMI was calculated between all node pairs in the model (all channel pairs of the EEG signals) for all temporal windows we defined above. We took an average across SMI values between pairs and defined the average SMI as the amount of total information sharing in each temporal window. For the EEG signals, we compared pairwise SMI values of real data to the pairwise SMI values of twenty surrogate data set with phase randomization technique and used only statistically significant SMI values (*p* < 0.01, Wilcoxon rank-sum test). The average SMI value of EEG channel pairs was used as an amount of total information sharing in each temporal window.

The network susceptibility was also measured in both model and EEG signals. The network susceptibility χ was defined as following (Yoon et al., 2015):


$$\chi =N\frac{<r^{2}\left(t\right)>-{<r\left(t\right)>}^{2}}{<r\left(t\right)>}$$

We defined the stationary dynamical susceptibility χ for each temporal window to see how susceptible the brain state would be to the external information from the outside of the brain in each temporal window.

### Topographic Similarity

To understand distinct functional network configurations engaged in low and high *R* windows, we measured topographic similarity *S^amp^*, which is defined as Spearman correlation between node degrees and node amplitudes.

In the model, the degree of brain regions was defined as the number of structural connections between one region and the other regions. The instantaneous amplitude *Z*(*t*) was measured by the absolute value of the complex variable *z*(*t*). The instantaneous Spearman correlation between degree and amplitude, *S^amp^*(*t*), was calculated for each time point for 60 s for all parameter combinations of the model. Then we took average of *S^amp^*(*t*) for each temporal window. For the EEG data, the degree of a node (channel) was inferred from the average functional connectivity strength over time measured by weighted phase lag index (wPLI) within a frequency range from 4 to 12 Hz for each subject. The wPLI is a measure that captures phase locking between two signals *X* and *Y*, relatively robust to volume conduction of EEG (Vinck et al., 2011).


$$wPLI_{XY}=\frac{\left|E\left\{I\left(C_{XY}\right)\right\}\right|}{E\left\{\left|I\left(C_{XY}\right)\right|\right\}}=\frac{\left|E\left\{\left|I\left(C_{XY}\right)\right|sgn\left(I\left(C_{XY}\right)\right)\right\}\right|}{E\left\{\left|I\left(C_{XY}\right)\right|\right\}}$$

where *I*(*C*_*XY*_) is an imaginary part of cross-spectrum *C*_*XY*_ between two signals *X* and *Y*. Here we used Hilbert-transformed complex signals for the calculation of cross-spectrum *C*_*xy*_. The *wPLI*_*XY*_ equals to 1 if the phases of one signal *X* always lead or lag the phases of the signal *Y*, and equals to 0 if the phase lead/lag relationship between two signals is perfectly random. We constructed the wPLI matrix across all channels for each 30-s epoch with a 5-s overlap and binarized the wPLI matrix for each epoch with the top 30 % wPLI values. We calculated the degree of channels for each epoch and took the average over all epochs so that we can capture the statistically intuitive structural degree that reflects the strength of neural communication across brain regions associated with each EEG electrode. Note that the degree is extracted from the CS (baseline) for each subject and applied to the analysis for the disrupted states of consciousness induced by isoflurane and ketamine. Since MCS and UWS patients have no baseline, we excluded those data set for this analysis. The instantaneous amplitude was calculated by the absolute value of the Hilbert transformed EEG signals from a frequency range of 4–12 Hz. The instantaneous Spearman correlation between degree and amplitude, *S^amp^*(*t*), was calculated for each subject and CS, ISO, and KET.

### Correlation Between Network Synchronization, Symbolic Mutual Information, Network Susceptibility, and Topographic Similarity

**FIGURE 2:**
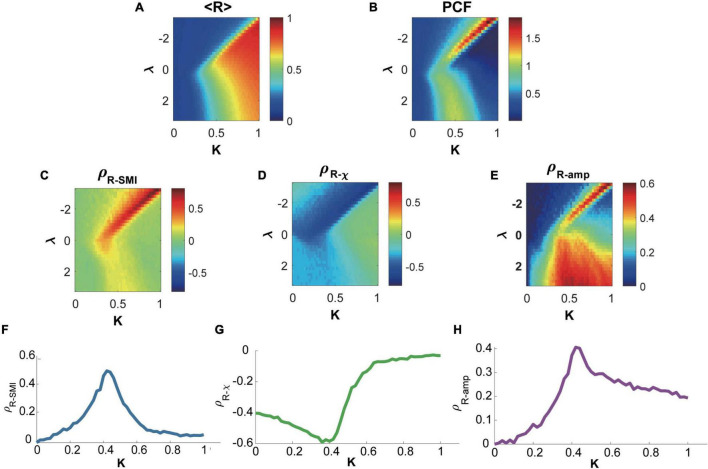
Relationships between network synchronization, symbolic mutual information, network susceptibility, and topographic similarity in the brain network model near and far from criticality. **(A)** The average network synchronization <R> across all parameter sets *K* and λ. **(B)** Pair correlation function (PCF), a surrogate indicator of criticality in parameter space *K* and λ Accordingly, a critical state is defined with the maximum PCF. Spearman correlations between network synchronization R and **(C)** symbolic mutual information SMI, **(D)** network susceptibility χ, and **(E)** topographic similarity *S^amp^*. The brain network model shows a maximum positive (negative) correlation between R and SMI (R and χ) near the critical states (states with maximum PCF along with *K* for each λ). The *S^amp^* also shows a maximum positive correlation with R near the critical states. A representative example for the correlation between **(F)** R and SMI, **(G)** R and χ, and **(H)** R and *S^amp^* relationship was investigated at λ = −0.6. The critical state appears *K*≅0.42.

According to our hypothesis, levels of network synchronization are associated with the network’s preference for internal and external modes in the brain on a sub-second time scale. Therefore, we calculated Spearman correlation between the level of network synchronization and the information processing metrics (SMI and χ). We also calculated Spearman correlation between the level of network synchronization and the topographic similarity. The correlation between *R*, *SMI*, χ, and *S^amp^* were calculated across all temporal windows. For the model, we calculated Spearman correlations for each parameter combinations with one hundred different simulations (Figure 2). For the EEG data, we calculated Spearman correlations between *R*, *SMI*, χ, and *S^amp^* for each subject and each state to investigate whether the relationships we found from the model hold for the empirical EEG data.

### Joint Histogram Between Symbolic Mutual Information and χ

To visualize the distinct network preferences for internal and external processing modes, a joint histogram of *SMI* and χ of low and high *R* windows was calculated. The joint histogram for the model was calculated near and far from the critical state across all temporal windows of one hundred frequency distributions with the bin size 0.02 for *SMI* and 0.1 for χ. For the EEG data, we calculated a joint histogram of SMI and χ for each temporal window with the bin size 0.02 for SMI and 0.1 for χ during CS, ISO, KET, PSY, MCS, and UWS. We can easily figure out the temporal window’s preference for internal or external information by calculating the joint histogram.

### Power-Law Analysis for Dwell-Time of the Positive Correlation Between Degree and Amplitude

It has been known that one of the characteristic features of criticality is the power-law distributions of dynamics. The dynamics following probability distribution *p*(*x*)∝*x*^−β^ imply that all values *x* can occur without a characteristic size or scale (Alstott et al., 2014). It has been also known that the frequency density of phase-locking intervals and the change in the number of phase-locked pairs between successive time points display power-law distributions at criticality in the Ising model and Kuramoto model (Kitzbichler et al., 2009). Since the network synchronization is correlated with the topographic similarity *S^amp^* (relationship between functional network configuration constrained by network structure in the brain), we estimated the probability distribution of dwell-time of *S^amp^* to check whether the relationship between functional network configuration and network structure follows power-law during conscious wakefulness. For the EEG data, we measured dwell-time of positive correlation periods (hub-dominant configuration periods) across all subjects for each state. We used a python package ‘‘power-law’’^1^ to estimate the probability distributions of positive correlation periods. We fitted dwell-time distributions to power-law and exponential distributions and compared their loglikelihood values to determine which distribution is well-fitted to the data. Using the loglikelihood ratio *R_L_* and p-value, we obtained the estimated probability distribution with the exponent β. *R_L_* has a positive value if the power-law fit is more appropriate, while it has a negative value if the exponential fit *p*(*x*)∝*e*^−β^ is more appropriate. We set the minimal value *x*_*min*_ as 200-ms for the EEG data to match the speed of the brain network dynamics and focused on a “heavy-tailed” characteristic of power-law.

### Functional Magnetic Resonance Imaging Experimental Protocol, Data Acquisition, and Preprocessing

The fMRI data were collected at two different research sites, Wisconsin and Shanghai. The experimental protocol for the first data set recorded from Wisconsin was approved by the Institutional Review Board of Medical College of Wisconsin (MCW). Propofol was administrated to 15 healthy volunteers (male/female 9/6; 19-35 years) and the OAAS (observer’s assessment of alertness/sedation) was quantified to measure the level of behavioral responsiveness. This dataset has been previously analyzed with a different hypothesis and published (Huang et al., 2020). In the current study, we used conscious states (baseline) that subjects responded readily to verbal commands (OAAS score = 5) and deep sedation that subjects have no response to verbal commands (OAAS score = 2–1). At the deep sedation level, the plasma concentration of propofol was maintained at equilibrium by continuously adjusting the infusion rate. The corresponding target plasma concentrations of propofol vary across subjects (1.88 ± 0.24 μ*g*/*ml*) due to the individual variability of anesthetic sensitivity. Total 14 subjects were analyzed in the current study because one subject had to be excluded due to excessive movements. Rs-fMRI data of the conscious state and deep sedation both consisted of a 15-min scan. Gradient-echo EPI images of the whole brain were acquired on a 3T Signa GE 750 scanner (GE Healthcare, Waukesha, Wisconsin, United States) with a standard 32-channel transmit/receive head coil (41 slices, TR/TE = 2000/25 ms, slice thickness = 3.5 mm, field of view = 224 mm, flip angle = 77°, image matrix: 64 × 64). Rs-fMRI was co-registered by high-resolution anatomical images. See (Huang et al., 2020) for a more detailed experimental protocol.

The second dataset includes 28 healthy participants (male/female 14/14) and 21 patients with disorders of consciousness (male/female 18/3). The study was approved by the Institutional Review Board of Huashan Hospital, Fudan University. The healthy controls had no history of neurological or psychiatric disorders or were taking any kind of medication. The patients were diagnosed as either minimally conscious or in the vegetative state/unresponsive wakefulness syndrome (UWS) according to the Coma Recovery Scale-Revised (CRS-R) on the day of fMRI scanning. We analyzed the data of 13 patients who were diagnosed as UWS in the current study. This dataset also has been published using different a hypothesis (Huang et al., 2020). Gradient echo EPI images of the whole brain for the second dataset were acquired on a Siemens 3T scanner (Siemens MAGNETOM, Germany) with a standard 8-channel head coil (33 slices, TR/TE = 2000/35 ms, slice thickness = 4 mm, field of view = 256 mm, flip angle = 90, image matrix: 64 × 64). Total two hundred EPI volumes (6 min and 40 s) were acquired with high-resolution anatomical images.

Preprocessing steps were implemented in AFNI^2^, which included slice timing correction, head motion correction/realignment, frame-wise displacement estimation, coregistration with high-resolution anatomical images, spatial normalization into Talaraich stereotactic space, high-pass filtering (>0.008 Hz), regressing out undesired components (e.g., physiological estimates, motion parameters), spatial smoothing (6 mm full-width at half-maximum isotropic Gaussian kernel), temporal normalization (zero mean and unit variance). Global signal regression (GSR) was not applied to preserve whole-brain patterns of co-activation or co-deactivation. More detailed preprocessing steps were described in the paper (Huang et al., 2020).

### Co-activation Pattern Analysis for Functional Magnetic Resonance Imaging Data

An unsupervised machine-learning approach named k-means clustering algorithm was used to define co-activation patterns (CAPs). The algorithm operates as a classifier of a set of objects (e.g., fMRI volumes) to minimize within category (e.g., patterns) differences and maximize across category differences. Specifically, the fMRI volumes were pooled together across states (baseline, propofol-induced states, ketamine-induced states, and UWS) and subjects, and classified into k number of clusters (patterns) based on their spatial similarity, yielding a set of CAPs or brain states (Huang et al., 2020). As such, we obtained time-series of discrete CAP labels from the original fMRI time-series (voxels × volumes). The volumes containing motion artifact tagged by motion censoring procedure were not included in the CAP analysis. We evaluated the validity of the number of clusters by measuring the inter-dataset similarity (Euclidean distance) of the average CAP occurrence rate distributions of conscious and unresponsive conditions. We searched the number of k from 2 to 30 using an index, (*CC* + *UU*)/(2 × *CU*), as the ratio of inter-dataset similarity among conscious conditions (CC) and among unresponsive conditions (UU) versus the inter-dataset similarity among conscious and unresponsive conditions (CU) across all datasets and found the optimized number of k as 8. Therefore, each fMRI volume from all datasets corresponded to one of the patterns from 8 CAPs. In the current study, we used pre-defined CAPs and analyzed a subset (*n* = 69) of originally published datasets including subjects under wakefulness baseline condition (*n* = 42), deep sedation induced by propofol (*n* = 14), and UWS patients (*n* = 13).

### Dominant Co-activation Patterns for Incoherent and Highly Synchronized States

In the EEG data, we defined the incoherent and highly synchronized state that presumably corresponds to externally susceptible/internally integrated state using certain thresholds of the level of synchronization. To define the incoherent/highly synchronized state for the fMRI data, we also measured the level of synchronization *r*(*t*) across voxels for each fMRI volume. Then we classified fMRI transient state to incoherent or highly synchronized state using the thresholds ${<r\left(t\right)>}_{total}\pm 0.5\sqrt{<r^{2}\left(t\right)>-{<r\left(t\right)>}^{2}}$. The < *r*(*t*) > _*total*_ were calculated from all concatenated fMRI volumes of three different states of consciousness (baseline, propofol-induced deep sedation, and UWS). Then we investigated occurrence rate distributions of CAPs for the incoherent or highly synchronized state with a permutation test. For the permutation test, we generated null CAP time-series by 20000 permutations, randomly and uniformly exchanging CAPs in time. Then the original fMRI time points (volumes) that levels of synchronization have below (above) threshold ${<r\left(t\right)>}_{total}-0.5\sqrt{<r^{2}\left(t\right)>-{<r\left(t\right)>}^{2}}$ (${<r\left(t\right)>}_{total}+0.5\sqrt{<r^{2}\left(t\right)>-{<r\left(t\right)>}^{2}}$) for each state of consciousness were selected and patterns corresponding to those time points were used to examine whether the occurrence probabilities significantly deviate from uniformly random sequences. The significantly dominant CAPs were determined at the significance level of *p* < 0.005 (0.5% percentile of the null distributions: one-sided).

## Results

### Criticality Produces Distinct Preferences for Internal Information and External Stimuli in the Brain Network: A Computational Model Study

We propose that criticality in the brain network produces distinct preferences for internal information of the network and external stimuli, which may result from the general property of the network responsiveness with high and low levels of global network synchronization. By contrast, the preferences may vanish when the brain network is positioned far from criticality. To test this hypothesis, we used a large-scale human brain network model, simulating various brain network behaviors near and far from criticality.

The coupled Stuart-Landau model, which has successfully described the characteristics of EEG, MEG, and fMRI in different states of consciousness, was used in this study. Spontaneous oscillations of the brain network were simulated with different sets of model parameters including the bifurcation parameter λ, coupling strength *K*, and diffusive coupling α (see “Materials and Methods” for details), which produces different brain states near and far from criticality. The level of network synchronization *R* was measured with a temporal average of the order parameter *r*(*t*). Figure 2A presents < *R* >, which is an average *r*(*t*) over 60 s, in the parameter space (λ, *K*). Figure 2B presents a pair correlation function (PCF) in the parameter space (λ, *K*). The PCF measures a temporal variance of network synchronization as a surrogate indicator of criticality (Yoon et al., 2015). The critical state was defined with the maximal PCF, indicating the largest synchronization fluctuation at criticality. For instance, we defined the brain state at *K*≅0.42 as one of the critical states in the model (Figure 2B). The brain network’s preferences for integrating internal information of the network and external stimuli were respectively evaluated with symbolic mutual information SMI (King et al., 2013) and network susceptibility χ (Yoon et al., 2015). SMI quantifies the amount of shared information and χ quantifies network susceptibility to external stimuli. Figures 2C,D show that both measures have maximal (minimal) Spearman correlation coefficients with *R* near the critical states. In other words, a brain network with a larger *R* has a larger *SMI* and smaller χ (preference for integrating internal information of the network), whereas a brain network with a smaller *R* has a smaller *SMI* and larger χ (preference for external stimuli). Here, *R*, *SMI*, and χ were calculated within each 250-ms temporal window with 50-ms overlap, and the correlations among them were calculated across the temporal windows. Note that the sub-second temporal scale is a suitable time resolution for the information integration processing in a large-scale network level (Bressler and Tognoli, 2006).

We also examined the functional network configurations of high and low *R* windows to investigate any structural difference in functional brain networks for two different preferences. We first calculated topographic similarity *S^amp^*, which is defined as a Spearman correlation between node degrees of the anatomical brain network and average amplitudes of the simulated brain activities. In this analysis, a larger *S^amp^* implies that hub nodes are more dominant in network dynamics along with higher amplitudes. Then we calculated the Spearman correlation coefficient between *S^amp^* and *R*. Figure 2E shows that the correlation between *R* and *S^amp^* is also maximal near the critical state, suggesting that a highly synchronized brain network is more likely to have a hub-dominant functional network configuration that is optimal for integrating internal information of the brain network. We present a representative example of the relationships at λ = −0.6 with the diffusive coupling of α = 0.5. Figures 2F–H show that those relationships are the largest near the critical state (ρ^*R*−*SMI*^ = 0.50 at *K* = 0.42, ρ^*R*−χ^ = −0.59 at *K* = 0.38, and *S^amp^* = 0.41, respectively). The results are consistent with different diffusive coupling parameters, α = 0 and α = 1 (Supplementary Figures 1, 2).

### The Preferences for Internal Information and External Stimuli of the Brain Network Are Prominent Near Criticality in a Sub-Second Time Window: A Computational Model Study

**FIGURE 3:**
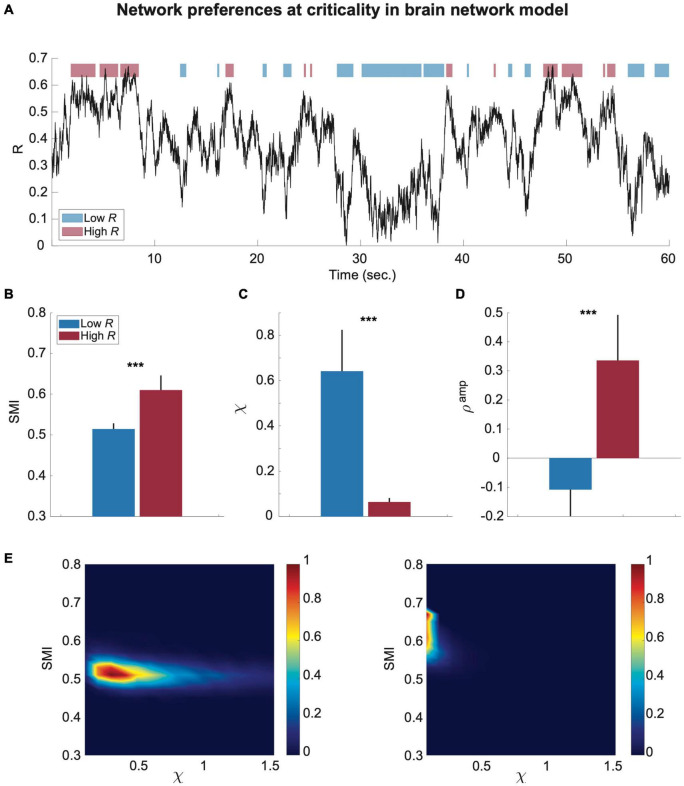
The preferences of the brain network for internal information integration and external responsiveness are prominent in sub-second time windows (250-ms) in the model. **(A)** An example of a network synchronization fluctuation near a critical state. Red and blue bars indicate temporal windows classified into low and high R. The SMI, χ, and *S^amp^* were calculated for each window. Comparisons of **(B)** SMI, **(C)** χ, and **(D)** *S^amp^* between temporal windows of low R (blue) and high R (red) are presented. The black lines of the colored bars indicate from 25% to 75% quantiles. A Wilcoxon rank-sum test was performed (***p < 0.001). **(E)** Joint histograms of the low (left) and high (right) R windows. The low R window is characterized by relatively low SMI and high χ, while the high R window is characterized by relatively high SMI and low χ.

To test whether the preferences of the brain network are significantly different in the time domain, we classified the simulated brain signals into low and high *R* windows and investigated *SMI* and χ values for each temporal window (250-ms). Figure 3A shows a temporal evolution of *R* during 60 s in the brain network model near a critical state at λ = 0.6. The low *R* and high *R* windows were defined with thresholds *R* < 0.3 and *R* > 0.5, respectively (blue: low *R* and red: high *R* in Figure 3A). The high *R* windows are characterized by large *SMI* and small χ, whereas the low *R* windows have relatively small *SMI* and large χ (^***^p < 0.001 for *SMI*; ^***^p < 0.001 for χ, Wilcoxon rank-sum test, in Figures 3B,C). The results show that the brain states of these high and low *R* windows define preferences for internal information vs. external responsiveness in the network. We also showed that high R windows have a large positive *S^amp^*; by contrast, low *R* windows have negative *S^amp^* (^***^p < 0.001, Wilcoxon rank-sum test, Figure 3D). The *S^amp^* value itself was variable across different parameter sets, but the positive correlation between *R* and *S^amp^* near criticality was consistent (Supplementary Figures 1, 2). Our results suggest that the fluctuation of network synchronization near criticality naturally produces a continuous switching between internal and external modes in the brain network with different functional network configurations. In Figure 3E, the joint histograms of *SMI* and χ (see “Materials and Methods” for more details) clearly show the distinct preferences of high (right) and low (left) *R* windows for internal information of the network and external stimuli (i.e., larger *SMI* and smaller χ for high *R* windows; smaller *SMI* and larger χ for low *R* windows), respectively. Notably, such typical preferences of high and low *R* windows only appear near criticality. We also compared the mean values of *R*, *SMI*, and χ of low and high *R* windows for three different states in the model (critical state *Cr*, below critical state *Cr_b_*, and above critical state *Cr_a_*); the results were presented in Supplementary Figure 3. The results show that the mean value of *R* is not linearly correlated with *SMI* and χ, rather it shows obvious state-dependence. For instance, for *Cr_a_*, the *SMI* of low *R* windows are larger than those of high *R* windows for *Cr* and *Cr_b_*, even though their *R* values are smaller. Such state dependence also holds true for χ.

### The Preferences of the Brain Network for Internal Information and External Stimuli Are Prominent in the Conscious State: An Electroencephalography Study

**FIGURE 4:**
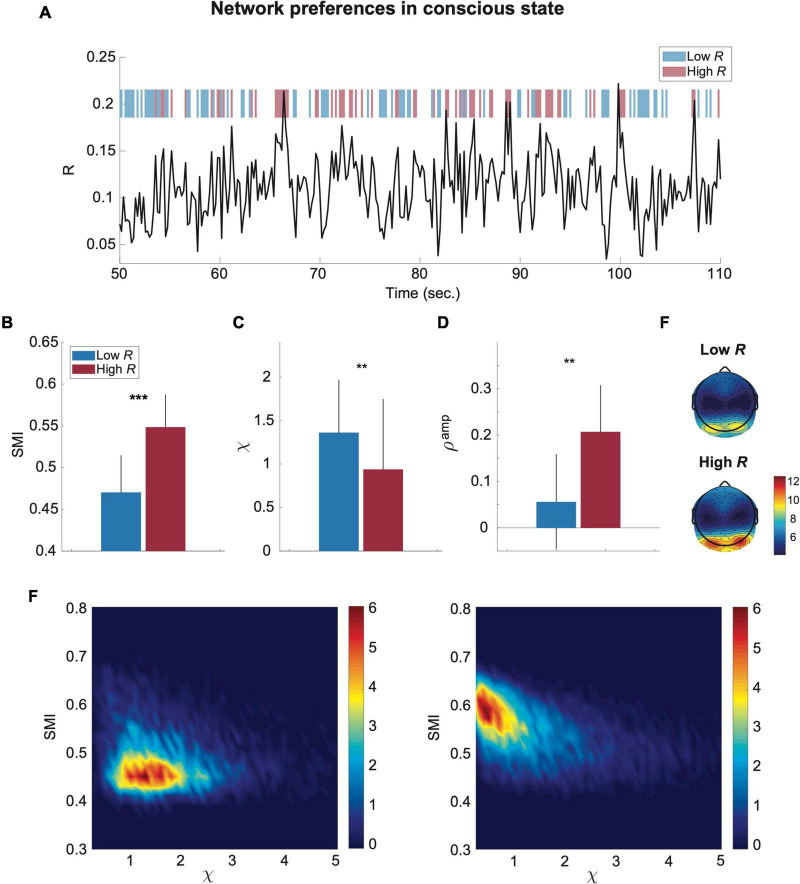
EEG functional brain networks in conscious state present distinct preferences for internal information integration and external responsiveness in the time domain. **(A)** An example of a network synchronization fluctuation during a conscious state. Red and blue bars respectively indicate temporal windows classified into low and high R. The SMI, χ, and *S^amp^* were calculated for each 250-ms window. Comparisons of **(B)** SMI, **(C)** χ, and **(D)** ρ^amp^ between low R (blue) and high R (red) windows are presented. The black lines of the colored bars indicate from 25% to 75% quantiles. A Wilcoxon rank-sum test was performed (***p < 0.001 and **p < 0.01). **(E)** Functional brain network configurations for low and high R windows. As expected in the model study, the amplitudes of the posterior area (hub regions) in high R windows are larger than those in low R windows. **(F)** Joint histograms of the temporal window of low (left) and high (right) R. The low R windows are characterized by relatively low SMI and high χ, while the high R windows are characterized by relatively high SMI and low χ. The empirical data are consistent with the model prediction.

To test the modeling results empirically, we analyzed EEG signals of conscious eye closed-resting states in humans (*n* = 24). Computational model and empirical studies suggested that theta and alpha-band (4–12 Hz) oscillations are globally networked in the cortex and spatiotemporally organize neural processing with traveling waves across the human cortex (Zhang et al., 2018; Roberts et al., 2019). Thus, here we focused on the EEG signals of 4–12 Hz that is suitable to test the characteristic behavior of the brain network near criticality. We also observed that the results of this frequency band (4–12 Hz) are most consistent with the model predictions (Supplementary Figure 4). As in the model data analysis, we calculated *R*, *SMI*, χ, and *S^amp^* for each temporal window of 250-ms. Figure 4A presents a temporal evolution of *R* for 60 s. We used thresholds $R={<R>}_{total}\pm 0.5\sqrt{<R^{2}\left(t\right)>-{<R\left(t\right)>}^{2}}$, where < *R* > _*total*_ is an average of *R* across all subjects and all states of EEGs (see “Materials and Methods” for more details), to classify the temporal windows into high *R* and low *R* windows (blue and red blocks in Figure 4A). Note that we used different thresholds for the source signals (model) and sensor signals (EEG) due to the differences in their signal variability. The results of the EEG analysis were consistent with the results of the computational model and show large *SMI* and small χ for high R windows and small *SMI* and large χ for low *R* windows (^***^p < 0.001 for *SMI*; ^**^p < 0.01 for χ, Wilcoxon rank-sum test, Figures 4B,C). The high *R* windows also presented a large positive *S^amp^* (^**^p < 0.01, Wilcoxon rank-sum test, in Figure 4D). For *S^amp^*, we used average degrees of wPLI networks and average amplitudes of EEG signals of a temporal window, assuming that the wPLI network averaged over a long time may resemble its underlying structural network (Kim et al., 2018). Similar to the brain network model near criticality, the brain network during conscious states demonstrates the distinct preferences of high and low *R* windows for internal information and external stimuli as well as the posterior hub-dominant network configuration in high *R* windows (Figure 4E). Furthermore, joint histograms of *SMI* and χ clearly visualize the distinctive preferences of high (right) and low (left) *R* windows in conscious states (Figure 4F). In sum, the brain network constructed from EEGs during conscious states shows the typical network properties of high and low *R* windows in terms of *SMI* and χ, which is predicted by the model study, and temporally yields continuous switching between preferences for internal information and external stimuli.

### The Preferences of the Brain Network for Internal Information and External Stimuli Vanishes in Different States of Consciousness: An Electroencephalography Study

**FIGURE 5:**
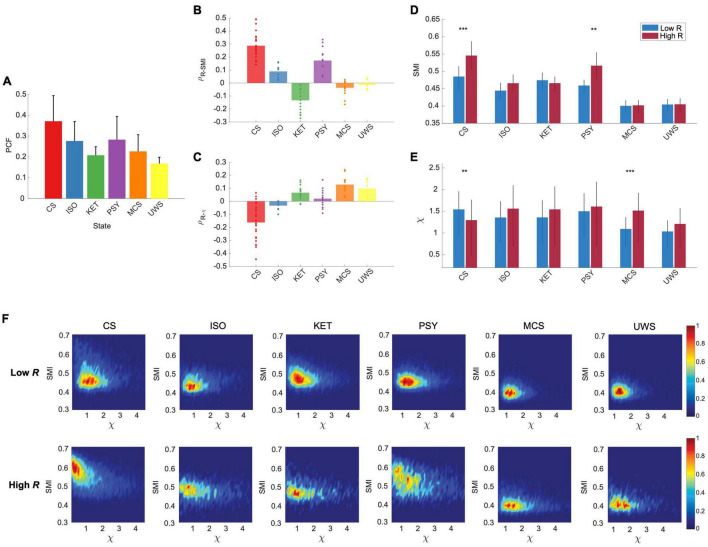
The preferences of the brain network for internal information integration in the network and external responsiveness are significantly disrupted in different states of consciousness (CS: conscious state, ISO: isoflurane-induced unconsciousness, KET: ketamine-induced unconsciousness, PSY: subanesthetic ketamine-induced psychedelic state, MCS: minimally conscious state, and UWS: unresponsive wakefulness syndrome). **(A)** PCF in different states of consciousness (Mean ± SD). The CS shows the largest PCF as expected in the brain network model. PCFs are significantly different among the states except for the ISO and PSY pair and the KET and MCS pair (*p <* 0.001, Kruskal-Wallis test with a multiple-comparison test using Tukey-Kramer method). Spearman correlations between **(B)** R and SMI and **(C)** R and χ in different states of consciousness. Dots indicate the correlation values of subjects. As expected in the model study, CS shows a maximum positive (negative) correlation between R and SMI (R and χ), and these relationships are disrupted in the different states of consciousness. **(D)** SMI and **(E)** χ for low and high R windows in different states of consciousness. Only CS shows significant differences between high and low R windows in both SMI and χ (***p < 0.001 and **p < 0.005; Wilcoxon rank-sum test). **(F)** Joint histograms of different states of consciousness. The distinct preferences of low and high R windows disrupt in all different states of consciousness (ISO, KET, PSY, MCS, and UWS).

Our modeling results predict that the distinct preferences of high and low *R* windows for internal information and external stimuli vanish when the brain network deviates from criticality, with reduced synchronization fluctuation (Figure 2). To test this model prediction, we analyzed EEGs from conscious eyes-closed resting states in humans (CS, *n* = 24), isoflurane-induced unconsciousness (ISO, *n* = 9), ketamine-induced unconsciousness (KET, *n* = 15), subanesthetic ketamine-induced psychedelic state (PSY, *n* = 15), and disorders of consciousness such as minimally conscious states (MCS, *n* = 16) and unresponsive wakefulness syndrome (UWS, *n* = 9). We first calculated the PCF to evaluate the synchronization fluctuation and demonstrated that the PCF significantly decreases in all altered and disrupted states of consciousness (*p* < 0.001, Kruskal-Wallis test, Figure 5A). The PCFs of the five different states of consciousness are significantly different from one another (*p* < 0.001, multiple-comparison test with Tukey-Kramer method) except for the ISO and PSY (*p* = 0.59) pair and the KET and MCS pair (*p* = 0.08).

We also calculated the *R*, *SMI*, and χ for each temporal window and calculated the correlations between them. With reduced PCFs, as expected, all of the altered and disrupted states of consciousness lost the correlations between *R* and *SMI* and between *R* and χ, except for *R* and *SMI* of PSY (Figures 5B,C). While investigating several other frequency bands (1–4 Hz, 1–20 Hz, 8–12 Hz, 13–30 Hz, and 30–42 Hz), we found that the frequency band of 8–12 Hz is also consistent with the results from 4 to 12 Hz (Supplementary Figure 3). Furthermore, we also classified the temporal windows of all states into high and low *R* windows with the same thresholds of CS. The distinct preferences between internal information and external stimuli observed in CS (large *SMI*/small χ for high *R* and small *SMI*/large χ for low *R* window) faded away in the different states of consciousness (*SMI*; CS: ^***^p < 0.001, ISO: *p* = 0.03, KET: *p* = 0.48, PSY: ^**^p < 0.005, MCS: *p* = 0.90, and UWS: *p* = 1, χ; CS: ^**^p < 0.005, ISO: *p* = 1, KET: *p* = 0.06, PSY: *p* = 0.10, MCS: ^***^p < 0.001, UWS: *p* = 0.14, Wilcoxon rank-sum test) (Figures 5D,E). We also found that the *SMI* of high *R* windows in CS is significantly reduced in ISO, KET, PSY, MCS, and UWS (*p* < 0.001; CS vs. all other states, one-way ANOVA with multi-comparison test with Tuckey-Kramer method, suggesting that disrupting the brain’s capability for processing internal information may be a common mechanism for inducing both altered and depressed states of consciousness. The χ of low *R* windows of ISO, KET, MCS, and UWS was significantly decreased or maintained compared to that of CS. In Figure 5F, joint histograms of *SMI* and χ visualize that only CS shows distinctive preferences of low and high *R* windows for internal information of the network and external stimuli. Furthermore, we investigated association of the posterior hub-dominant structure in the various states of consciousness (Supplementary Figure 5). Different from CS, ISO shows anterior dominant network configuration for the high R windows and KET shows no difference between low and high *R* windows. However, similar to CS, PSY presents the posterior hub-dominant structure in high *R* windows. Notably, the significant network preference for internal integration with the posterior hub-dominant configuration only occurred in CS and the altered state of consciousness (PSY) associated with consciousness (Supplementary Figure 5), implying that the brain’s capability for internal information integration with the specific network configuration might play a more prominent role in maintaining consciousness than network susceptibility.

### Dominant Functional Magnetic Resonance Imaging Co-activation Patterns at High and Low Levels of Synchronization in Conscious and Unconscious States

Finally, we tested whether the preference of high and low *R* windows can be confirmed using a different brain imaging modality such as fMRI BOLD signals, investigating an association with well-known fMRI networks that are activated while the brain carries out tasks requiring internal and external information processing. We predicted that the fMRI networks relevant to internal or external information processing may be preferentially activated in high or low R windows and such preferences may change in disrupted states of consciousness.

**FIGURE 6:**
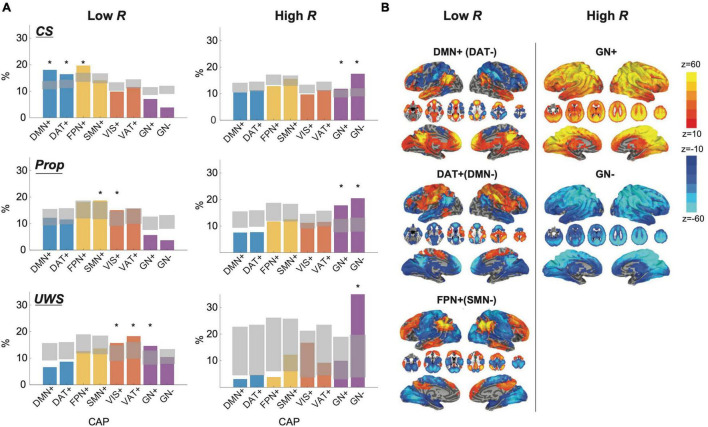
Dominant fMRI co-activation patterns (CAPs) for low and high R windows in conscious states (CS), propofol-induced unconsciousness (Prop), and unresponsive wakefulness syndrome (UWS). **(A)** Occupancy percentages of CAPs in CS (1st row), Prop (2nd row), and UWS (3*^rd^* row) for low R (left) and high R (right) windows. Gray areas indicate quantiles ranging from 0.5% to 99.5% from the distributions of 20000 permutation sets. Significantly dominant CAPs are marked as ‘*’ in the figure. The CAPs consist of eight different functional networks such as a default-mode network (DMN+), dorsal attention network (DAT+), frontoparietal network (FPN+), sensory and motor network (SMN+), visual network (VIS+), ventral attention network (VAT+), and a global network of activation and deactivation (GN+ and GN−). Functional networks including DMN+(DAT−), DAT+(DMN−), and FPN+(SMN−) are dominant in low R windows, while functional networks including GN+ and GN- are dominant in high R windows in CS. **(B)** Spatial maps of CAPs for low and high R windows in CS. Spatial maps of other CAPs are presented in the Supplementary Figure 7.

To determine which fMRI networks are dominant in high and low *Rs*, we first classified human fMRI BOLD signals from conscious states (CS, *n* = 42), propofol-induced unconscious states (Prop, *n* = 14), and UWS (*n* = 13) into low and high *R* windows with the thresholds $R_{th}={<R>}_{total}\pm 0.5\sqrt{<R^{2}\left(t\right)>-{<R\left(t\right)>}^{2}}$, where < *R* > _*total*_ is an average network synchronization across all subjects and all states. We then investigated the occurrence rates of co-activation patterns (CAPs) for the low and high *R* windows, comparing them with the occurrence rates of a null CAP time-series generated by 20,000 permutations of BOLD signals (Figure 6A). The CAPs were defined as 8 typical patterns of BOLD co-activation across voxels such as default mode network (DMN+), dorsal attentional network (DAT+), frontoparietal network (FPN+), sensory and motor network (SMN+), visual network (VIS+), ventral attention network (VAT+), and a global network of activation and deactivation (GN+ and GN−). More detailed explanation of the CAP analysis is in “Materials and Methods” and Supplementary Figure 7. According to our hypothesis, the low *R* windows should be characterized by networks that are sensitive to external stimuli, whereas the high *R* windows should be more relevant to networks that are insensitive to external stimuli. Our results show that in CS the low *R* windows are dominated by DMN+, DAT+, and FPN+, which are sensitive to external stimuli (Sadaghiani et al., 2015), and the high *R* windows are dominated by GN+ and GN−, which are known as arousal networks that are insensitive to external stimuli (Figure 6B; Chang et al., 2016; Liu et al., 2018; Turchi et al., 2018). The dominant CAPs in CS were replaced with other CAPs in Prop and UWS, mostly deactivating DMN+. Thus, the distinct preferences of high and low *Rs* for internal information of the brain network and external stimuli observed near criticality in the conscious state can be confirmed with fMRI BOLD signals. The disrupted preferences were also observed universally in the brain network that distant from criticality and the EEG and fMRI data in different states of consciousness.

## Discussion

With a computational model, we demonstrated that when the brain network resides near a critical state, the network synchronization *R* correlates with the amount of shared information (*SMI*) within the network and the network susceptibility (χ) to external stimuli, respectively. The highly synchronized states prefer internal information integration within the network (large *SMI* and small χ), while the incoherent states prefer external stimuli (small *SMI* and large χ). However, the correlations between *R*, *SMI*, and χ were diminished when the brain network deviated from criticality. The modeling study also showed that a highly synchronized brain network displays predominant hub activities, providing a network condition favorable for internal information integration. The modeling results were compared with empirical data across diverse states of consciousness. First, the brain network during conscious states exhibited the largest synchronization fluctuation compared to the different states of consciousness (ISO, KET, PSY, MCS, and UWS), which confirms criticality of the conscious brain. Second, the EEG functional brain networks during CS showed temporally fluctuating preferences between the internal information of the network and external stimuli, presenting a large *SMI*/small χ at high *R* and a small *SMI*/large χ at low *R*. These preferences were significantly diminished in the different states of consciousness. Additionally, the dwell-time of the hub-dominant network configurations at high *R* windows were organized in a scale-free manner. The power-law distribution of the dwell-time changed into a random organization (i.e., exponential distribution) in unconscious states (Supplementary Figure 8). Finally, the most frequently observed CAPs at high (low) *R* in the fMRI signals corresponded to internally integrative (highly susceptible) brain states.

In sum, distinct network preferences for the internal information within the network or external stimuli spontaneously emerge when the brain network is positioned near criticality and in conscious states. The brain loses such distinct preferences far from criticality and in different states of consciousness (ISO, KET, PSY, MCS, and UWS), which results in a disruption of temporal information processing capability.

### Criticality’s Novel Role in Conscious Brain Function

Biological systems can obtain many functional benefits from operating near a critical point of a phase transition. Criticality in biological systems, conjectured to emerge as the result of adaptive and evolutionary processes, produces an optimal balance between stability and instability, optimal computational capacity, large dynamical repertoires, and greater sensitivity to stimuli (Beggs, 2008; Kitzbichler et al., 2009; Tagliazucchi et al., 2012; Haimovici et al., 2013; Cocchi et al., 2017; Kim and Lee, 2019). The brain near criticality should display characteristic features that are empirically measurable: maximal sensitivity, large spatiotemporal correlations, and large variances in synchronization. Maximal sensitivity is an important property for sensory systems in the brain, such as the olfactory, visual, and auditory systems, optimizing responses to environmental cues (Chialvo, 2006; Kinouchi and Copelli, 2006; Bushdid et al., 2014; Hudspeth, 2014; Shew et al., 2015). A large spatiotemporal correlation is crucial for the brain to induce coordinated neural activities across space and time (Tagliazucchi et al., 2012), which is a useful mechanism for the generation of long-lasting and slow-decaying memories at multiple timescales (Deco and Jirsa, 2012). A large variance of synchronization induces large statistical complexity and large repertoires in the brain, which is due to the maximal variety of attractors and metastability (Haldeman and Beggs, 2005; Deco and Jirsa, 2012; Haimovici et al., 2013) in corresponding state spaces. It facilitates the spontaneous generation of complex patterns required for optimizing the brain’s capability for storing and processing information, enabling the brain to constantly traverse different network configurations, which is associated with cognitive flexibility. Based on our findings in the model study and the empirical data analyses, we propose a novel role for criticality in brain function, which is the provision of a platform that enables the brain to rapidly transition between distinct temporal preferences for internal information within the network and external stimuli. The large network synchronization fluctuation that spontaneously emerges near criticality enables the brain network to integrate internal information or be sensitive to external information in the time domain. Furthermore, the temporally variable network preferences might play the role of a functional platform for continuous switching between internal and external information modes in the brain, which is essential for constructing inner models of the outside world through the recursive learning process (Honey et al., 2017) as well as for the emergence of consciousness. In addition, as the network becomes distant from a critical state along with reduced synchronization fluctuation, such distinct preferences vanish as altered and depressed states of consciousness occur. Our model simulation and empirical data analyses explicitly demonstrate that the variance of synchronization (PCF) is maximal at a critical state (Figure 2B) and in conscious states (Figure 5A). By contrast, the variance is significantly diminished in different states of consciousness such as general anesthesia, psychedelic experiences, and pathological disorders of consciousness (Figure 5A).

### Brain Network Preferences Vanish in Different States of Consciousness

In different states of consciousness (ISO, KET, PSY, MCS, and UWS), brain networks no longer possessed distinct preferences for the internal information of the network or external stimuli. It is important to consider how brain networks in different states of consciousness lose such preferences and what differentiates altered vs. depressed states of consciousness. Decreased *SMI*, especially in high *R* windows, is a common feature across altered and depressed states of consciousness regardless of the type of anesthetic or traumatic injury. However, network susceptibility χ was relatively unchanged across different states (ISO, KET, PSY, MCS, and UWS). These results imply that the brain network preferentially loses its capability to integrate internal information, while its susceptibility to external stimuli remains relatively intact. In other words, the brain network may be able to receive external stimuli, but it cannot be globally integrated during different states of consciousness, which may be specifically related to functional deafferentation and disconnection from the external world due to the isolation of the thalamocortical network (Mhuircheartaigh et al., 2013). In addition, loss of consciousness accompanied the disruption of the posterior hub-dominant network configuration (Supplementary Figure 5), which impairs the functional role of hubs for integrating and transmitting information within the hierarchical brain network (Stam and van Straaten, 2012; van den Heuvel and Sporns, 2013). This hub disruption also causes the selective inhibition of top-down processes by preferentially impeding information flow from hub nodes to peripheral nodes in the brain network (Marinazzo et al., 2012; Stam and van Straaten, 2012; Cohen et al., 2018). Numerous studies have demonstrated that anesthetics selectively inhibit higher-order information integration in top-down processes while preserving the bottom-up and primary sensory processes, highlighting the importance of top-down processes for the emergence of consciousness (Liu et al., 2012; Schroter et al., 2012). Our model and empirical data analyses suggest that such preferential inhibition of internal information integration in the brain network, specifically, via the disruption of the hub-dominant network configuration in high *R* windows, which is likely associated with high-order information integration, is a characteristic phenomenon that can occur when a complex network deviates from criticality. If the network moves to states far from criticality (super- or subcritical states), it will preferentially hamper the normal functions of hubs that have dense connections.

The altered and depressed states of consciousness were associated with distinctively impaired network properties, *SMI* and χ, in high and low *R* windows, all of which differentiate these states. Interestingly, the psychedelic and minimally conscious states showed opposite network properties (Figure 5D). The psychedelic state preserved the network preference for integrating the internal information of the network (i.e., positive correlation between *R* and *SMI*); however, the network preference for susceptibility to external stimuli between high and low *R* windows vanished. The low *R* windows no longer retained the bias toward external stimuli. In contrast, the MCS lost the capability for integrating internal information (i.e., it exhibited a small *SMI* in a high *R* window), but instead presented larger network susceptibility in high *R* windows, which is the opposite compared to consciousness (Figure 5E). In other words, the brain network of the MCS disrupted the network’s capability for internal information integration with an abnormal relationship between network synchronization and susceptibility. Conversely, the brain network of the psychedelic state still functioned for internal information integration, however, it did not respond to external stimuli properly, which suggests an impaired network preference for external stimuli. Finally, we found that the dwell-time for the hub-dominant network configuration (a positive correlation between node degree and amplitude; *S^amp^* > 0) follows a power-law distribution while conscious but follows an exponential distribution during ISO and KET (Supplementary Figure 8). Considering the association of a hub-dominant network configuration (*S^amp^* > 0) with high *R* and *SMI* in both critical and conscious states (Figures 3, 4), the power-law of the dwell times implies that when the brain integrates internal information (higher *SMI*) in high *R* windows, the hub structure dominates the integration process in the brain network. Also, the posterior hub-dominant brain network configuration is organized temporally in a scale-free manner, which enables the brain to efficiently process higher-order information integration at various time scales. In contrast, the anesthetized states (ISO and KET) lose the brain’s capacity to process internal information (low *SMI* and *R*) and their temporal organization becomes close to random processes. More specifically, even with similar levels of *R*, the *SMI* values in high *R* windows are different across states, revealing a state dependence of the brain in relation to its capability for internal information integration in high *R* windows and emphasizing the significance of functional brain network configurations (Supplementary Figure 6).

### Dominant Functional Magnetic Resonance Imaging Co-activation Patterns at High and Low Levels of Synchronization

Functional magnetic resonance imaging studies have demonstrated that conscious states are correlated with critical dynamics (e.g., see Tagliazucchi et al., 2016). Dominant fMRI co-activation patterns for incoherent and synchronized windows in conscious states were presumably associated with brain networks that are sensitive and insensitive to external stimuli, respectively. We showed that, in conscious states, the co-activation patterns for incoherent windows were dominated by DMN+, DAT+, and FPN+. These networks have complex interactions and support higher-order cognitive functions. For example, the DMN engages in a variety of processes such as autobiographical memory, imagination, and self-referencing (Raichle, 2015). The DAT mediates cognitive processes such as goal-driven attention, inhibition, and top-down guided voluntary control (Corbetta and Shulman, 2002; Vossel et al., 2014). The FPN+ is known to flexibly alter its functional connections dynamically according to current task demands (Cole et al., 2013) and has a strong association with working memory (Murphy et al., 2020). Furthermore, these three networks are at a high position of a representational hierarchy, relatively far from the sensory and motor systems in terms of both functional connectivity and anatomical distance (Margulies et al., 2016). Such a hierarchical disposition is thought to allow these networks to process transmodal information in a way that is unconstrained by immediate external stimuli. We suggest that these spatially segregated co-activation patterns (DMN+, DAT+, and FPN+) may be associated with high sensitivity to stimuli. As supported by a previous study by Sadaghiani et al. (2015), pre-stimulus brain states with higher modularity (i.e., higher spatial segregation and lower global integration) bias toward detecting external stimuli, whereas pre-stimulus brain states with lower modularity (i.e., higher spatial homogeneity and higher global integrity) bias toward misses.

In contrast, we found that highly synchronized states during consciousness are dominated by GN+ and GN−. The two co-activation patterns are correlated with global EEG synchronization in the alpha frequency band (Liu et al., 2018) and associated with arousal fluctuations regulated by subcortical-cortical connectivity (Chang et al., 2016; Liu et al., 2018; Turchi et al., 2018). Particularly, it has been suggested that GN+ is specific to lapses in alertness associated with a transition to a state of lower arousal (Liu et al., 2018), rendering the neural system less sensitive to external stimuli. Taken together, our results suggest that the distinct preferences of the brain network for internal information or external stimuli in low and high levels of network synchronization, regardless of imaging modalities, are common network properties near critical states.

Notably, the temporal scales of the network preference switching in EEG and fMRI data are inherently different due to the measurement of different proxies of brain activity (EEG: neuronal processing; fMRI: blood-oxygenation level). Because of the lack of time resolution of fMRI data, we focused on identifying the relevant brain network structures in high and low *R* windows. However, since the fMRI signals near and far from criticality can be modeled by networked oscillators (Deco et al., 2017, 2018), we expect the general relationship between *SMI*, *R*, and χ near criticality we found with the networked canonical oscillator model also holds for the fMRI signals. Further study will be needed to confirm this.

### Potential Mechanisms for the Emergence of Distinct Preferences in the Brain Network

recent studies of brain network characteristics near criticality have provided some evidence of the potential mechanism for the emergence of continuous switching between internal and external modes. One of the potential mechanisms is a global fluctuation in neural gain mediated by ascending neuromodulatory nuclei such as the pontine locus coeruleus (Lee and Dan, 2012; Shine et al., 2018). A series of studies has suggested that the modulation of neural gain with the dynamic changes in noradrenaline results in a large fluctuation between network integration and segregation in the brain (Shine et al., 2018; Li et al., 2019). When the neural gain dynamics (presynaptic afferent input) of the locus coeruleus reside near criticality, a small fluctuation produces a sharp transition between network integration and segregation, which respectively accompanies optimized information transfer and optimized information storage in the brain (Li et al., 2019). Network integration and segregation have also been associated with an increased spatial correlation (correlated with elevated information transfer) and increased autocorrelation times (correlated with increased information storage) with correspondence to high and low levels of phase synchronization (Shine et al., 2018; Li et al., 2019).

A previous computational model study from our research team found that brain network responsiveness significantly depends on the level of network synchronization as well as the distance from criticality when a stimulus is applied (Kim and Lee, 2020). Based on these results, we suggested that a potential mechanism for the relationship between brain network synchronization and responsiveness is the phase response curve in physics, which is a general property of networked oscillators ubiquitously observed in physical and biological systems. The phase response curve describes the way that a system with a collective periodic behavior (for instance, circadian rhythms, cardiac rhythms, and spiking neurons) responds to external stimuli (Granada et al., 2009; Hannay et al., 2015). The response of an oscillating system is measured by the phase shift from the original phase. This phase shift (i.e., advancing or delaying the original phase) is an inherent characteristic of the oscillatory system, which is determined by given network configurations. Previous analytic studies discovered that a low (high) phase synchronization induces a large (small) phase response to a stimulus, proving that stimulating the phases around a stable fixed point of the phase response curve increases phase response, whereas stimulating the phases around an unstable fixed point decreases phase response (Hannay et al., 2015). These properties generally hold for networks with different coupling functions, network structures, and connectivity (Levnajić and Pikovsky, 2010).

The present study advances previous results by analyzing the role of synchronization fluctuations in the brain network, modulating the distance from criticality in the human brain network model and analyzing the empirical data from EEG and fMRI for various states of consciousness. We directly calculated the amount of information sharing (*SMI*) and network susceptibility (χ) with the network synchronization *R* in the time domain for both the model and data. We propose the large synchronization fluctuation is a functional platform for creating temporal windows that process internal or external information. In particular, the temporal windows characterized by large *SMI* and small χ play a role in internal information integration while defending against interventions from the outside world. These characteristics of a highly synchronized network create temporal windows shielded from external perturbations and simultaneously enable the brain to integrate globally distributed information across brain regions. Empirically, we demonstrated that such selective roles for temporal windows, observed in human brain networks in conscious states, are disrupted in different states of consciousness (ISO, KET, PSY, MCS, and UWS). Notably, in our model study, we did not consider the neuromodulation system, which implies that the temporal windows for internal or external information processing can arise through the interplay between regional brain activities while the interactions are close to criticality. In other words, the emergence of the network preference for internal or external information and the continuous fluctuation between these two modes originate as a generic network feature near criticality, regardless of the specific neurobiology.

### Limitations

There are several limitations in this study. First, our brain network model simulated source signals of the brain network, not the EEG signals themselves in conscious and unconscious states. Instead, we focused on identifying the generic features of brain networks near and far from criticality to study the alteration of the brain network preference for internal/external modes in conscious and unconscious states. Second, we quantitatively evaluated the preferences for internal information and external stimuli without external stimulation. Since we cannot directly measure the response of unconscious subjects for cognitive tasks, which is beyond the scope of the current study, quantitative evaluations were limited to investigating the relationship between *R*, *SMI*, and χ at a system level. However, we expect that the results would be similar to the model results where we have already shown the relationship between *R* and responsiveness with external stimuli (Kim and Lee, 2020). Third, we focused on 4–12 Hz because this frequency band demonstrates global network features in conscious states that are suitable for the application of our brain network model (Zhang et al., 2018). However, how the EEG of 4–12 Hz is globally networked in the cortex and the relevant neuroanatomy is still unclear. Furthermore, higher frequency oscillations—which might be important for information processing—were excluded. Fourth, in our previous model study, we found more specific amplitudes and phases of oscillators that determine large and small responsiveness to external stimuli. However, in this study, we only used the level of synchronization as a criterion to classify the temporal windows due to the complexity of the application to the EEGs of various states of consciousness. Fifth, we limited our fMRI analysis to the comparison of co-activation patterns between high and low *R* windows due to the small data length for measuring critical dynamics in fMRI data. Future studies should directly compare the relationships between *R*, *SMI*, and χ in both EEG and fMRI data with longer fMRI data.

## Conclusion

Based on both a computational model and empirical data analyses from EEG and fMRI, we propose a novel role for criticality in facilitating continuous switching between internal and external modes in the brain. We found that a large network synchronization fluctuation that emerges near criticality creates continuous switching between distinct network preferences for internal information and external stimuli. Specifically, we found high *SMI* and posterior hub dominant network configuration in high *R* windows, which naturally occur near criticality, play essential roles in maintaining consciousness in conscious states and psychedelic states. The distinct network preferences at high and low levels of synchronization that we found with a canonical networked oscillator model at criticality and EEGs during conscious states supported our argument together with the results of the fMRI co-activation pattern analysis and the EEG analysis of different states of consciousness (ISO, KET, PSY, MCS, and UWS). The computational model study and empirical data analyses lead us to propose that criticality creates a functional platform for network transitions between internal and external modes in the brain, which presumably play an essential role in constructing models of the self and the world.

## Data Availability Statement

The datasets presented in this article are not readily available because participants in these empirical studies did not give consent to have data made publicly available. Requests to access these datasets should be directed to UL, uclee@umich.edu.

## Ethics Statement

The studies involving human participants were reviewed and approved by the University of Michigan Medical School Institutional Review Board, Institutional Review Board of Medical College of Wisconsin, and Institutional Review Board of the Huashan Hospital, Fudan University. The patients/participants provided their written informed consent to participate in this study.

## Author Contributions

UL, MK, and HK designed the research. MK and HK simulated the model. MK analyzed the data. GM, ZH, DJ, and RI curated the data. GM, UL, MK, HK, and ZH wrote the manuscript. All authors reviewed the manuscript.

## Conflict of Interest

The authors declare that the research was conducted in the absence of any commercial or financial relationships that could be construed as a potential conflict of interest. The reviewer AM-W declared a past co-authorship with one of the authors GM to the handling editor.

## Publisher’s Note

All claims expressed in this article are solely those of the authors and do not necessarily represent those of their affiliated organizations, or those of the publisher, the editors and the reviewers. Any product that may be evaluated in this article, or claim that may be made by its manufacturer, is not guaranteed or endorsed by the publisher.

## Footnotes

1
https://pypi.org/project/powerlaw/

2
http://afni.nimh.nih.gov/
