# Supplementary material for: Criticality Creates a Functional Platform for Network Transitions Between Internal and External Processing Modes in the Human Brain
Source: Front Syst Neurosci. 2021 Nov 25;15:657809. doi: 10.3389/fnsys.2021.657809 (PMC8657781; doi:10.3389/fnsys.2021.657809)
Supplement: Supplementary file 1 [file Data_Sheet_1.PDF]

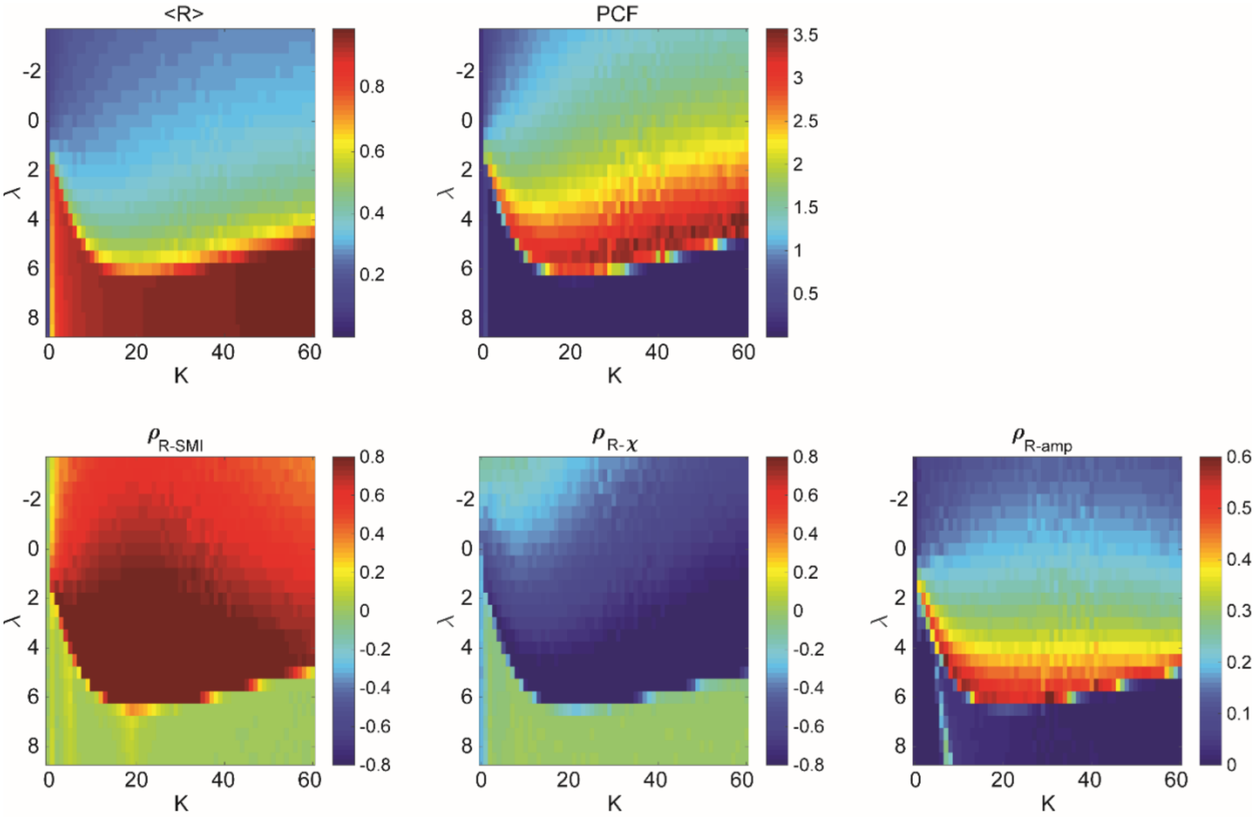

Figure S1. A level of global network synchronization *R* over 60 seconds, pair correlation function (PCF), and Spearman correlation between *R* and *SMI*, *R* and χ, and *R* and ρ^{amp} of the brain network model with the diffusive coupling strength $\alpha = 1$. (coupled in perfectly diffusive way). The critical states are defined as the states with the maximum PCF. The brain network model shows maximum positive (negative) correlations between *R* and *SMI* (*R* and χ) near critical states. The topographic similarity ρ^{amp} shows maximum positive correlations with *R* near the critical states. The results were calculated from the average of 100 different initial frequency configurations.

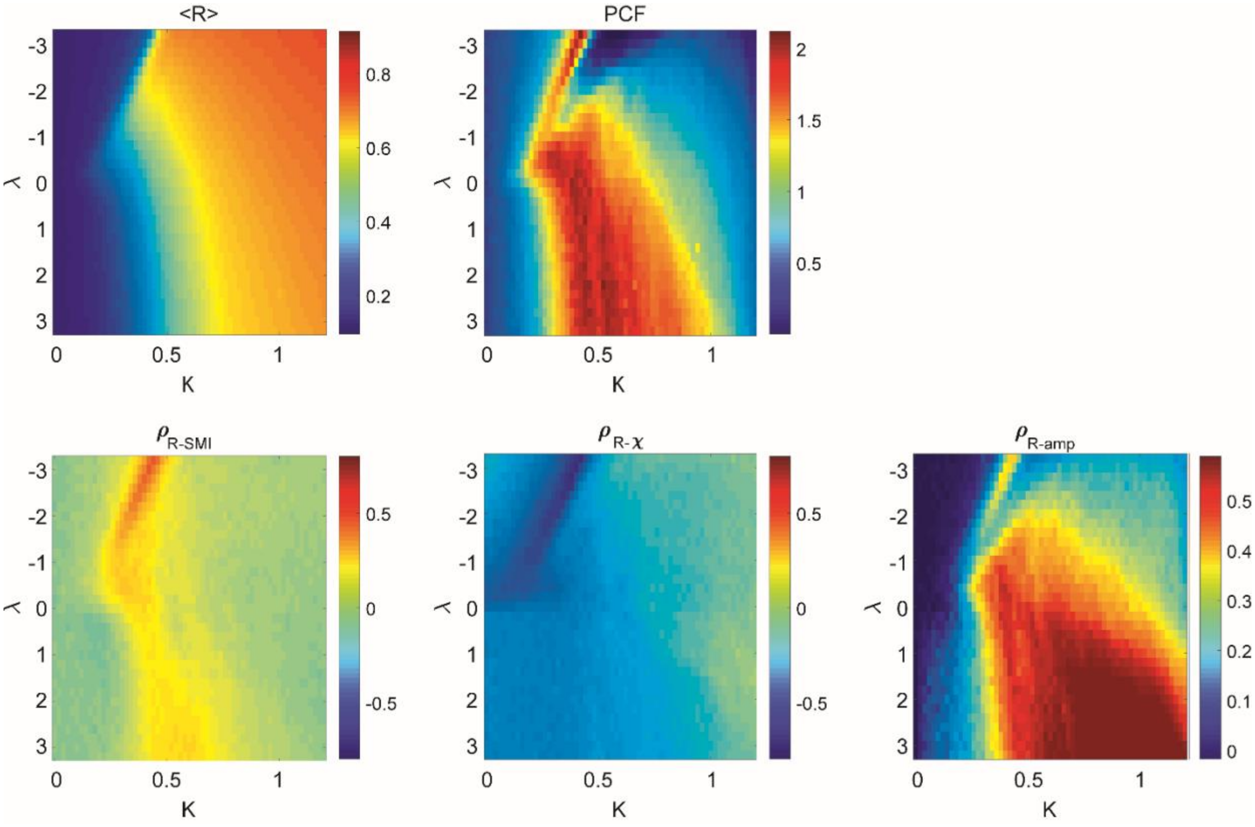

Figure S2. A level of global network synchronization *R* over 60 seconds, pair correlation function (PCF), and Spearman correlation between *R* and *SMI*, *R* and χ, and *R* and ρ^{amp} of the brain network model with the diffusive coupling strength $\alpha = 0$. (coupled in perfectly direct way). The critical states are defined as the states with the maximum PCF. The brain network model shows maximum positive (negative) correlations between *R* and *SMI* (*R* and χ) near critical states. The topographic similarity ρ^{amp} shows maximum positive correlations with *R* near the critical states. The results were calculated from the average of 100 different initial frequency configurations.

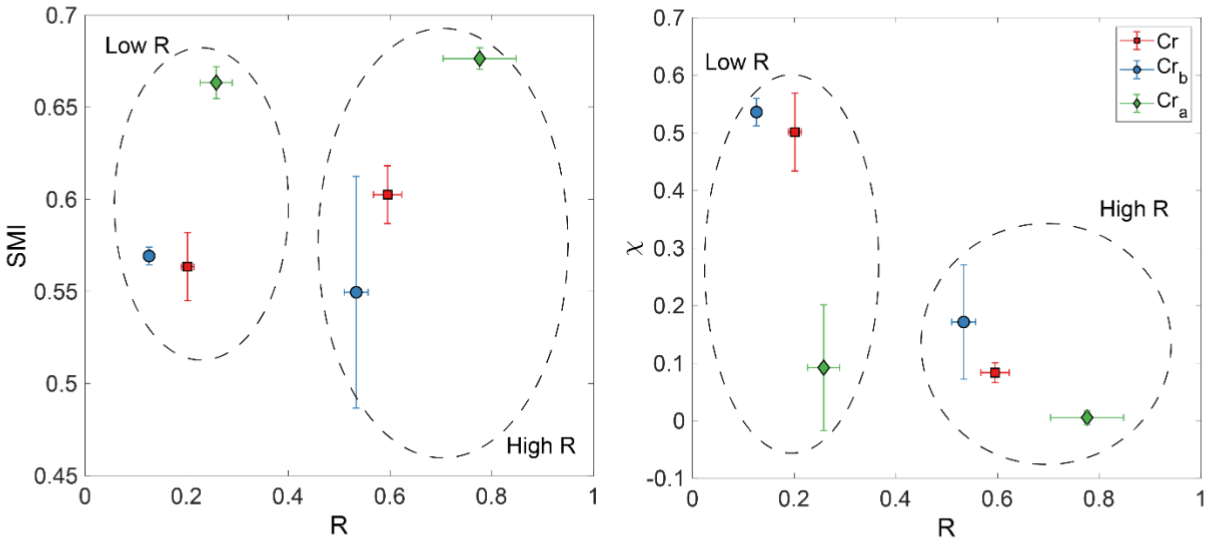

Figure S3. Comparisons of average *R*, *SMI*, and χ values at states near criticality (Cr), below criticality (Cr_b), and above criticality (Cr_a). Dashed circles indicate temporal windows of low *R* (high *R*). Error bars indicate SD. We took an average of the variables across all K and λ parameters of each state. Here Cr (red) shows significant *SMI* and χ differences between low and high *R* windows. The Cr_b (blue) shows low *SMI* but different χ values between low and high *R* windows. The Cr_a (green) shows high *SMI* and low χ for both high and low *R* windows, suggesting there might be the states stuck in high *SMI*. Please note that this is the average feature so that more detailed analysis should be performed in the future study.

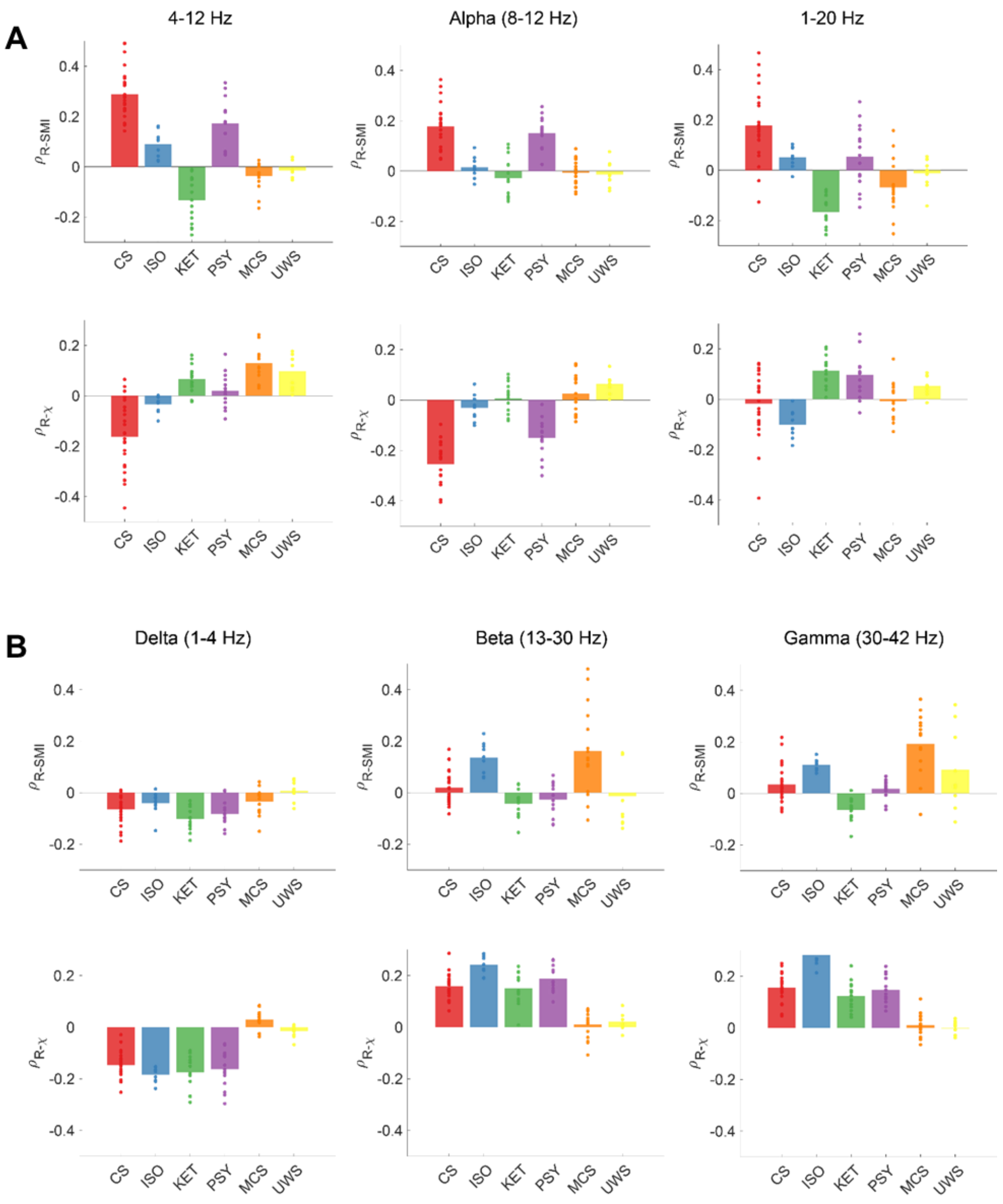

Figure S4. Spearman correlations between *R* and *SMI* (upper panels) and R and χ (lower panels) in different states of consciousness with different frequency ranges. Dots indicate the correlation values of subjects. (A) The relationships we found from the frequency range of 4-12Hz are maintained in alpha frequency range (8-12 Hz), but not in the frequency range 1-20 Hz. (B) The relationships we found from the frequency range of 4-12Hz are not maintained in the frequency range 1-4 Hz (delta), 13-30 Hz (beta), and gamma (30-42 Hz). It suggests that the relationships between global network synchronization and brain network's preferences for internal and external information processing could be a general property of globally networked oscillators in a frequency range from theta to alpha (4-12 Hz).

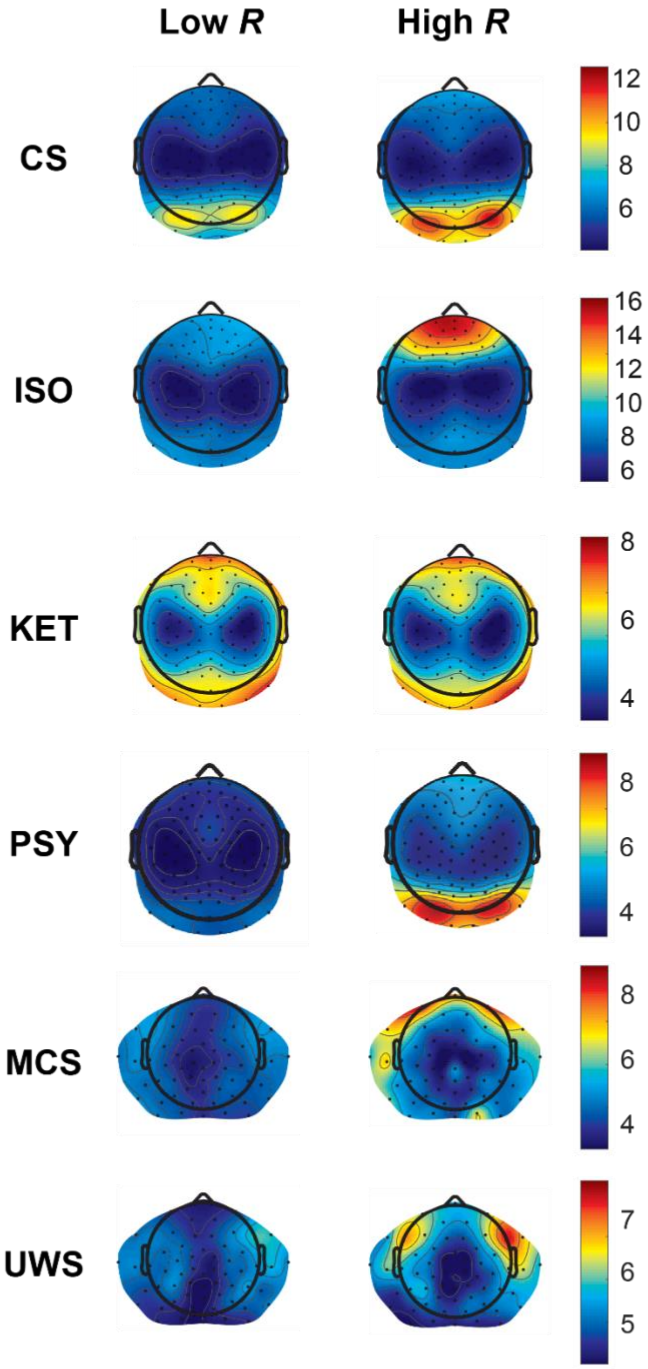

Figure S5. Functional brain network configurations of temporal windows of low and high *R* in different states of consciousness. During CS (baseline), the amplitudes of posterior area (hub regions) in high R windows are larger than the amplitudes in low R windows, with the positive ρ^{amp} for high R windows.

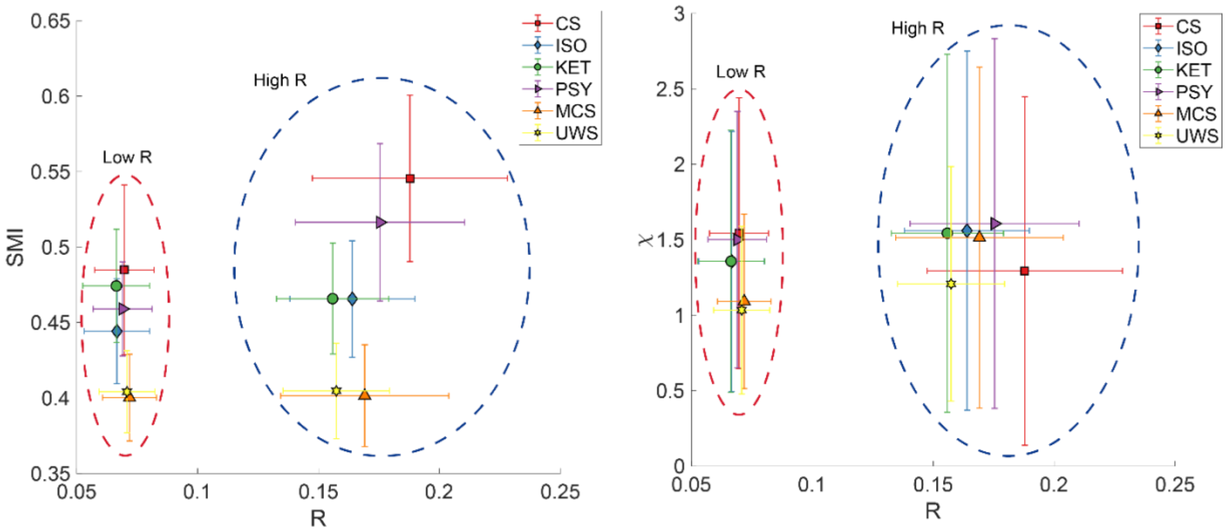

Figure S6. Comparisons of R, SMI, and χ values across different states of consciousness. Red (blue) dashed circles indicate temporal windows of low R (high R). Error bars indicate SD. The R values across all states are similar to each other for low and high R windows, respectively. The SMI values in high R window are different to each other, suggesting the amount of shared information in the brain network is different across the states even with the similar levels of R, suggesting the functional network configurations of the brain network are also important for internal information integration.

CAPs (co-activation patterns)

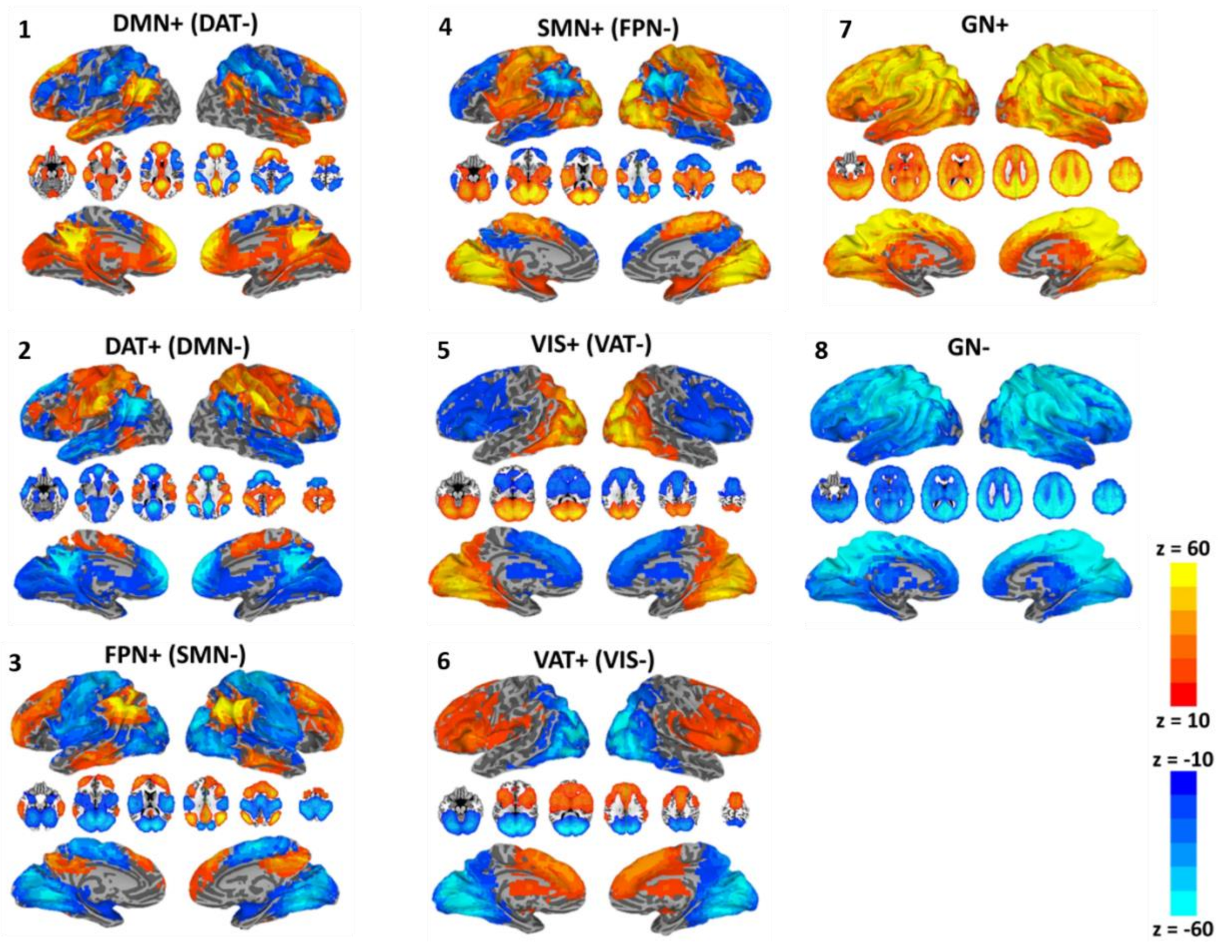

Figure S7. Spatial maps of fMRI co-activation patterns (CAPs). The CAPs consist of eight different functional networks such as a default-mode network (DMN+), dorsal attention network (DAT+), frontoparietal network (FPN+), sensory and motor network (SMN+), visual network (VIS+), ventral attention network (VAT+), and global network of activation and deactivation (GN+ and GN-). The figure is adapted with permission from the reference (60) Z. Huang, J. Zhang, J. Wu, G. A. Mashour, A. G. Hudetz, Temporal circuit of macroscale dynamic brain activity supports human consciousness. *Sci. Adv.* **6**, 87–98 (2020).

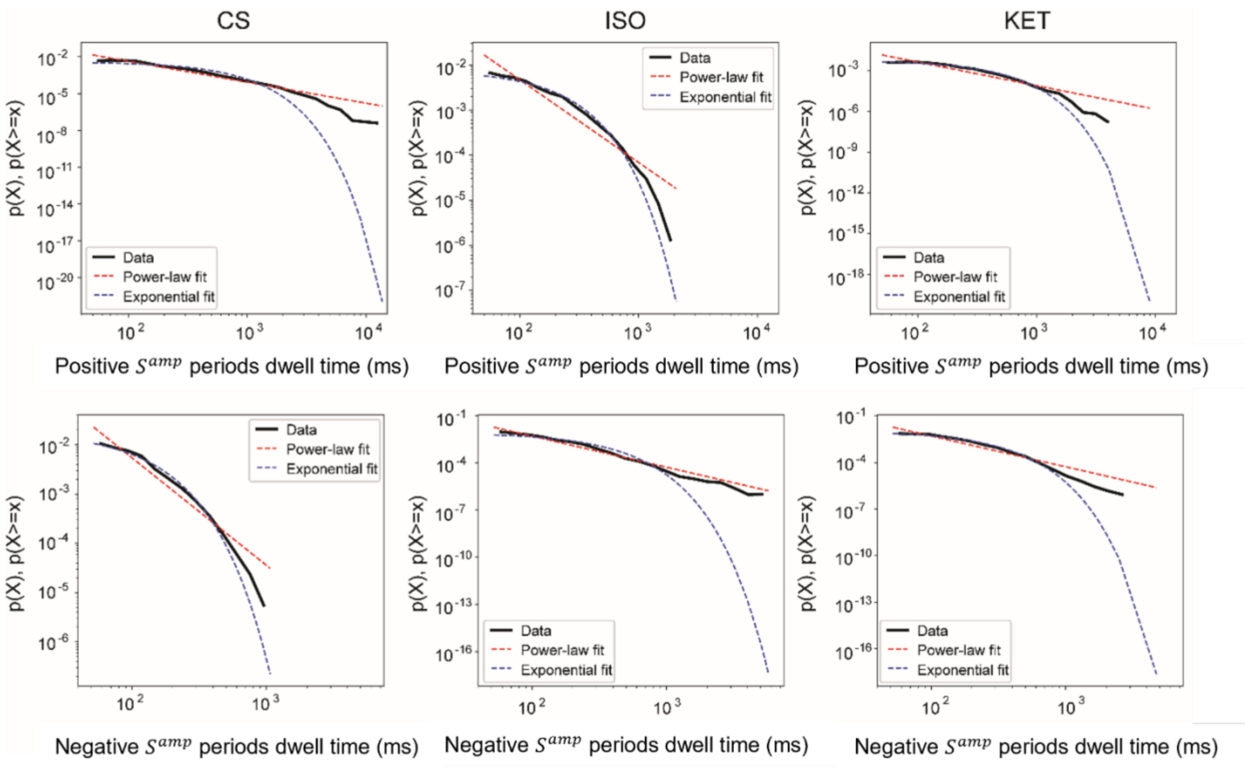

Figure S8. Probability density functions of S^{amp} dwell-time and fitted power-law and exponential distributions in CS, ISO, and KET. A dwell-time of positive (negative) S^{amp} periods was calculated across all subjects. The upper panel shows positive S^{amp} dwell-time in CS (left), ISO (middle), and KET (right). Black lines indicate the data, red (blue) lines indicate power-law (exponential) distribution-fitted lines. The positive S^{amp} in CS follows power-law, while that in ISO and KET is more likely to follow exponential distribution. The lower panel shows negative S^{amp} dwell-time in CS, ISO, and KET.
